# TREM2-mediated microglial phagocytosis of inhibitory synapses contributes to prolonged FS-induced epileptogenesis

**DOI:** 10.1038/s41420-026-03118-7

**Published:** 2026-04-11

**Authors:** Xiaoqian Wang, Hua Zhou, Yujie Zhai, Yunrong Zhang, Yao Cheng, Yi Yuan, Wenxiao Cao, Anqing Gao, Mengshuang Liu, Qiaoyun Wang, Hongliu Sun

**Affiliations:** School of Pharmaceutical Sciences, Binzhou Medical University, Yantai, China

**Keywords:** Epilepsy, Long-term depression, Inhibition-excitation balance

## Abstract

Febrile seizures (FS) are common convulsive episodes in childhood and an important etiological component in epilepsy. However, most currently available antiepileptic drugs cannot prevent epileptogenesis and may even exacerbate it. Triggering receptor expressed on myeloid cell 2 (TREM2)-mediated microglial phagocytosis of inhibitory synapses may play a pivotal role in epileptogenesis; however, the role of TREM2 in FS-induced epilepsy remains unclear. We established a Sprague-Dawley rat model of juvenile prolonged FS to analyze the associated molecular changes, epileptic susceptibility, and seizures. Our results confirmed that prolonged FS resulted in increased TREM2 levels, excessive phagocytosis by activated microglia targeting inhibitory synapses, and elevated epileptic susceptibility and seizures. Administration of a CD33 agonist (monosialoganglioside 1, GM1), a negative moderator of TREM2 that reduces its levels, attenuated microglial phagocytosis of inhibitory synapses and weakened susceptibility to epilepsy and seizures. The inhibitory effects of TREM2 knockdown were similar to those of CD33 activation. Blocking the outward-facing region of phosphatidylserine (PtdSer) to prevent TREM2 recognition resulted in increased TREM2 levels and deteriorated microglial activation. Finally, although vesicular GABA transporter (VGAT) levels were higher in the prolonged FS rats treated with annexin V, susceptibility to epilepsy and seizures were aggravated. This study revealed that reducing TREM2 levels may inhibit prolonged FS-induced epileptogenesis by alleviating the phagocytic function of activated microglia targeting inhibitory synapses, while preventing TREM2 from recognizing PtdSer has the opposite effect.

## Background

Febrile seizures (FS) are one of the most common types of seizures in children, generally occurring between 6 months and 5 years of age [1], with an incidence of 2%–5% [2]. Additionally, FS have a high incidence, are often accompanied by long-term sequelae, such as nerve damage, and can even progress to epilepsy, thus representing an important indicator of its development [3, 4]. Currently, there are many treatment options for FS, most of which involve reducing the overexcitability of brain tissues using antiseizure drugs [5]. However, most antiepileptic drugs cannot prevent the development of epilepsy and may even aggravate the neurological deficits caused by FS. Therefore, in-depth studies on the pathogenesis of FS is expected to play a crucial role in the development of therapies that inhibit the progression of FS to epilepsy.

FS and epilepsy are hyperexcitatory disorders [6, 7]. Experimental studies have confirmed that hyperexcitability, due to an impaired balance between excitability and inhibition, is a pathological feature of epilepsy induction [8–10]. Synapses are the basic units in the formation of neural networks [11]. Abnormal synaptic connections between neurons can lead to an imbalance in excitation/inhibition, which is the structural and functional basis of the formation of hyperexcitatory neural circuits, ultimately leading to the development of epilepsy [12, 13]. Furthermore, the development of epilepsy is often accompanied by an increase in excitatory synapses and a decrease in inhibitory synapses [14]. Microglia play an important role in this process by pruning and clearing synapses [15, 16]. Studies have confirmed a close relationship between microglia and the development of epilepsy, and that high inhibitory synapse phagocytosis and excitatory/inhibitory function imbalance result in abnormal neural network function [17, 18]. For instance, animal studies on the epileptic status induced by electrical stimulation have shown that microglial activation in the hippocampal dentate gyrus, the key site of epileptic network formation, and a significant reduction in the number of inhibitory synapses, result in abnormal neural network functional connectivity and excitation/inhibition imbalances [18]. In the brains of mice with epilepsy induced by Toxoplasma gondii infection, microglia in the hippocampus and neocortex are activated, and phagocytosis of inhibitory synapses results in weakened neural inhibitory function, which constitutes the structural basis of epileptic seizures in the later stages of infection [19]. In the brains of rats with pilocarpine-induced epilepsy, mossy fibers project to the dentate gyrus and CA3 region, enter the inner molecular layer of the dentate gyrus and CA1 region, establish abnormal synaptic connections, and induce epileptic seizures [20]. Repeated attacks induce excessive excitation of hippocampal granule cells, further damaging the function of intermediate inhibitory neurons and aggravating the imbalance between neuronal excitation/inhibition functions [20]. Therefore, we hypothesized that activation of phagocytic inhibitory synapses by microglia may be involved in FS-induced epilepsy.

The triggering receptor expressed on myeloid cell 2 (TREM2) plays a key role in the regulation of synaptic phagocytosis in microglia [21]. TREM2 is specifically expressed on the surface of myeloid cells and acts as a transmembrane receptor on the surface of microglia in the central nervous system [21]. Increased TREM2 expression is a key factor in the overactivation of phagocytic synaptic function in microglia. For example, studies in animals exposed to ethanol for a long time have confirmed that TREM2 is a key molecule mediating synaptic clearance in microglia; increased expression of TREM2 promotes the activation of microglia, greatly enhances the function of phagocytic synapses, and results in abnormal neural network structure and brain function defects. Moreover, TREM2 expression knockdown reverses microglial activation and phagocytic synapses, resulting in the repair of brain functional networks [22]. Studies on growth and development in young mice have also confirmed that TREM2 is key to synaptic pruning and the formation of brain functional networks. When TREM2 was knocked down, the number of activated microglia in the brains of the animals was significantly reduced, synaptic pruning function was weakened, and synaptic density was significantly higher than normal [23]. Studies of sleep deprivation also revealed that TREM2 upregulation in the hippocampus and other areas activated microglia, resulting in excessive phagocytosis of synapses in these areas and decreased synaptic density, ultimately resulting in neural network damage in the context of learning and memory; furthermore, the phagocytic synaptic function of microglia was significantly reversed when TREM2 levels were decreased, and as TREM2 levels continued to rise, phagocytic synapses were strengthened and neural network damage worsened [24]. Cell-based experiments confirmed that the phagocytic synaptic function of microglia is TREM2-dependent. When normal microglia were co-cultured with hippocampal neurons, the synaptic density of neurons decreased significantly, whereas when microglia derived from mice with TREM2 gene defects were co-cultured with hippocampal neurons, no significant change in synaptic density was observed [25]. Both animal models and cell-based experiments have confirmed that TREM2 is a key molecule that induces microglia to phagocytize synapses and plays a crucial role in neurosynaptic network defects in various neurological diseases [21].

Therefore, we speculated that TREM2 might play a key role in the progression of FS to epilepsy by activating the synaptic phagocytosis of microglia targeting inhibitory synapses, resulting in an imbalance in excitatory/inhibitory activity in the brain.

## Results

### Increased TREM2 levels, changed synapses and epileptic seizures in the brains of animals with prolonged FS

**Fig. 1:**
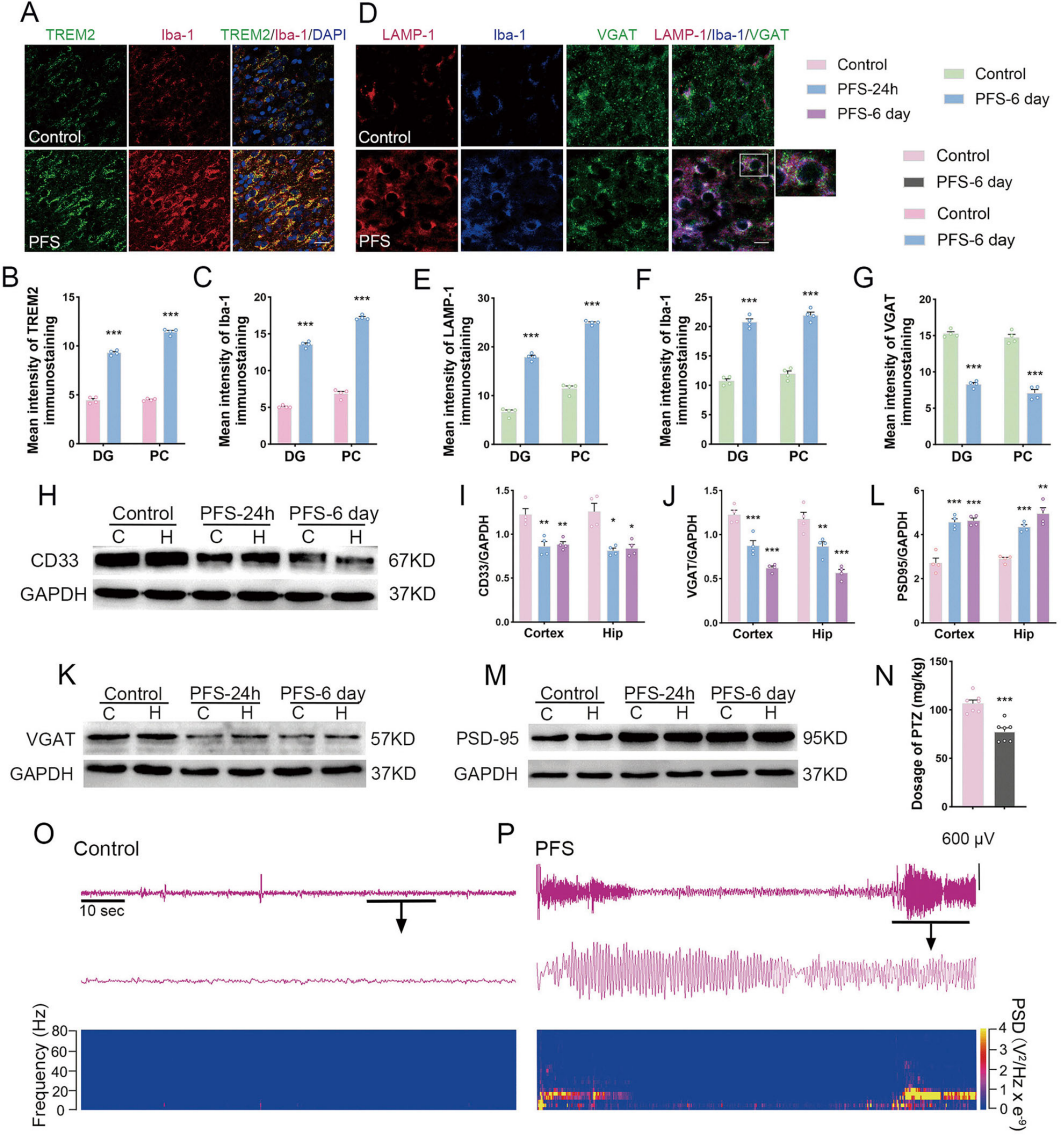
The changed levels of TREM2, CD33, and microglial phagocytosis and epileptic seizures after prolonged FS. **A**–**C** Immunohistochemical results of TREM2 (green) and Iba-1 (red) at 6 days after prolonged FS (scale bar = 25 μm, *n* = 4/group). Blue is DAPI. **D**–**G** Immunostaining localization analysis of LAMP-1 (red), Iba-1 (blue) and VGAT (green) at 6 days after prolonged FS (scale bar = 15 μm, *n* = 4/group). **H**, **I** Gray-scale bands and their normalized gray-scale values of CD33 relative to GAPDH in the hippocampus and cortex at 24 h and 6 days after prolonged FS (*n* = 4/group, One-way ANOVA). The gray-scale bands of VGAT (**J**, **K**) and PSD95 (**L**, **M**) relative to GAPDH in the hippocampus and cortex (*n* = 4/group). **N** The threshold dose of PTZ at 6 days after prolonged FS (*n* = 7/group, unpaired *t*-tests). **O**, **P** The representative EEG and PSD analysis spectra of the model rats at 6 days after prolonged FS. Mean ± SEM were presented. *p < 0.05, **p < 0.01, ***p < 0.001 vs. control group (unpaired *t*-tests).

Immunostaining localization analysis indicated increased levels of TREM2 (hippocampus, e.g., dentate gyrus (DG), *p* < 0.001; cortex, e.g., piriform cortex (PC), *p* < 0.001; Fig. 1A, B) and Iba-1 (Fig. 1A, C) in the brains of animals with prolonged FS compared with those in the control group (Supplementary Figs. 1 and 6). The fluorescence intensity of the lysosomal marker LAMP-1 (hippocampus, e.g., DG, *p* < 0.001; cortex, e.g., PC, *p* < 0.001; Fig. 1D, E) also increased, accompanied by an increase in activated microglia (Fig. 1D, F and Supplementary Figs. 1, 7). Meanwhile, the levels of the inhibitory synapse marker VGAT decreased significantly (Fig. 1D, G), and the levels of CD33, a negative regulator of TREM2, decreased (Fig. 1H, I). These results suggest enhanced phagocytic function of microglia with increased TREM2 levels.

Similar to the immunohistochemistry results, the levels of the inhibitory synaptic marker VGAT were significantly decreased (Fig. 1J, K), whereas those of the excitatory synaptic marker PSD95 were significantly increased (Fig. 1L, M). The susceptibility of the experimental animals to epilepsy was tested on the 6th day after FS [26, 27]. The results showed that the injected PTZ dose required to induce generalized epileptic seizures was significantly lower in the prolonged FS group than in the control group (*p* < 0.001; Fig. 1N), indicating increased epileptic susceptibility after prolonged FS. Representative EEG and power spectrum density (PSD) are shown in Fig. 1O, P.

### Increased TREM2 in M1 and M2 type microglia in the brains of animals with prolonged FS

**Fig. 2:**
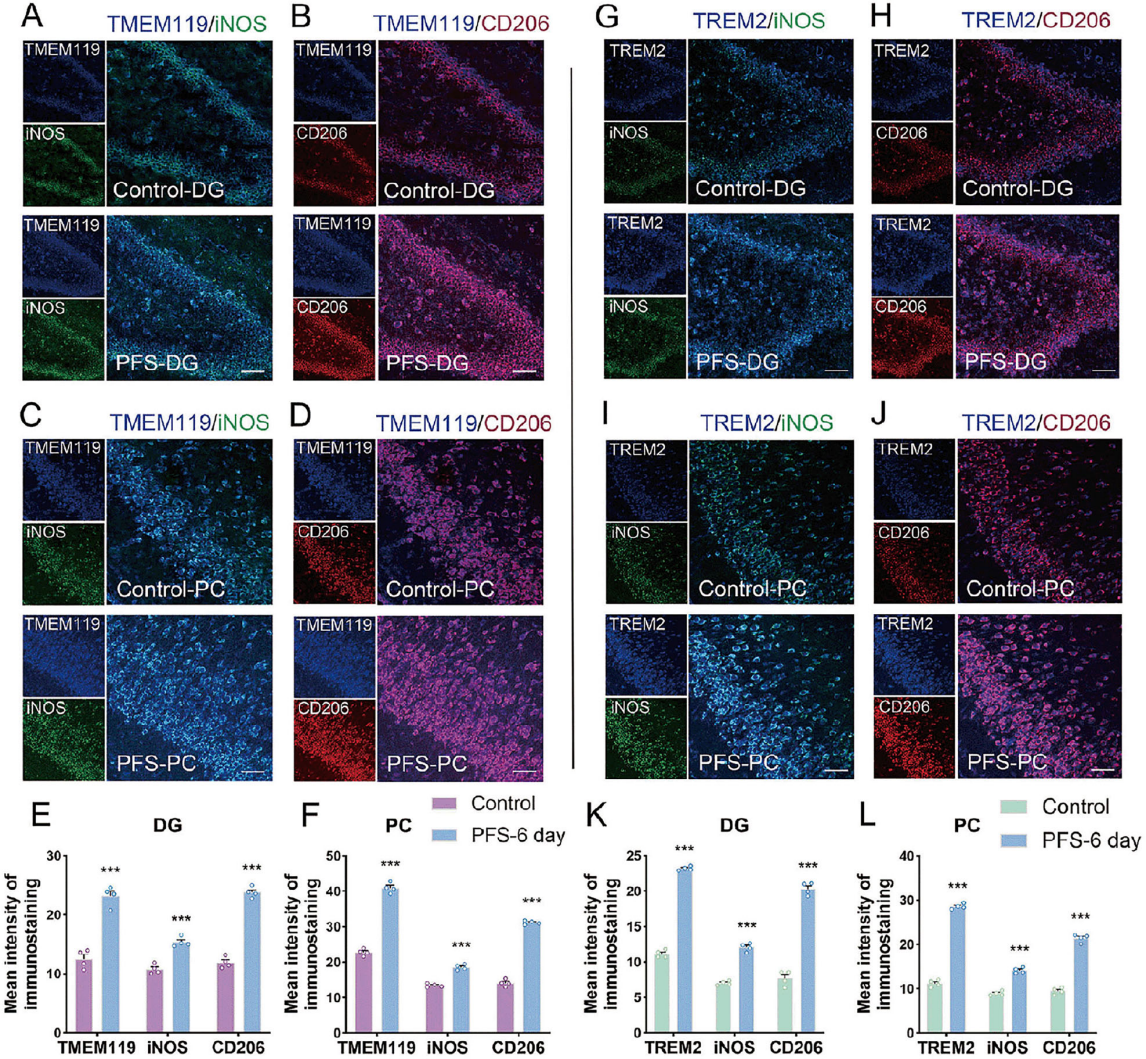
Dual immunofluorescence staining results of TREM2 and the M1/M2 activated microglia states in rats with prolonged FS. The immunohistochemical images (**A**–**D**) and analysis (**E**, **F**) of TMEM119 (blue) and M1 type (iNOS, green), and M2 type (CD206, red) at 6 days after prolonged FS. **G**–**L** The immunohistochemical results of the changed levels of TREM2 (blue marker) and M1 type (iNOS, green), and M2 type (CD206, red). Scale bar = 50 μm, *n* = 4/group. Mean ± SEM were presented. ***p < 0.001 vs. control group (unpaired *t*-tests).

Dual immunofluorescence results showed that the levels of the microglial marker TMEM119 were elevated after prolonged FS (Fig. 2A–F), with increased levels of co-localized iNOS (an M1-type microglia marker) in the hippocampus and cortex (Fig. 2A, C, E, F) and CD206 (an M2-type microglia marker, Fig. 2B, D–F). Further investigation of the distribution of TREM2 in M1 and M2 microglia in the hippocampus and cortex showed that it was significantly increased in both M1 and M2 microglia after prolonged FS (Fig. 2G–L).

### Changed levels of TREM2 in M1 and M2 type microglia after intervention targeting TREM2 or covering PtdSer with annexin V

**Fig. 3:**
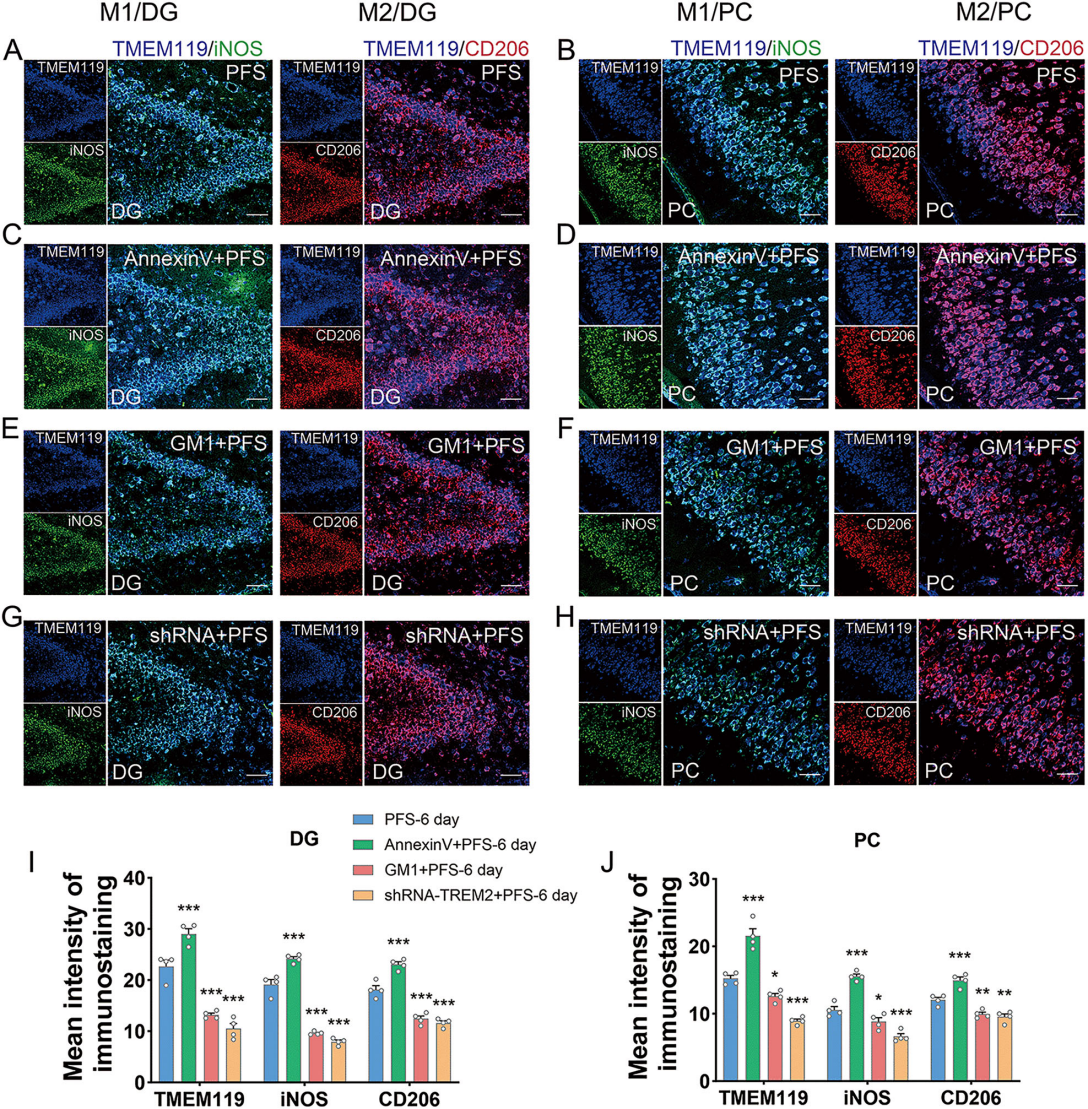
Dual immunofluorescence staining results of the M1/M2 activated microglia states after intervention in prolonged FS rats. **A**–**D** The immunohistochemical images of TMEM119 (blue) and M1 type (iNOS, green), and M2 type (CD206, red) at 6 days after AnnexinV intervention in prolonged FS. The immunohistochemical images (**E**–**H**) and analysis (**I**, **J**) of TMEM119 (blue) and M1 type (iNOS, green), and M2 type (CD206, red) at 6 days after GM1 and shRNA intervention in prolonged FS. Scale bar = 50 μm, *n* = 4/group. Mean ± SEM were presented. *p < 0.05, **p < 0.01, ***p < 0.001 vs. control group (one-way ANOVA).

**Fig. 4:**
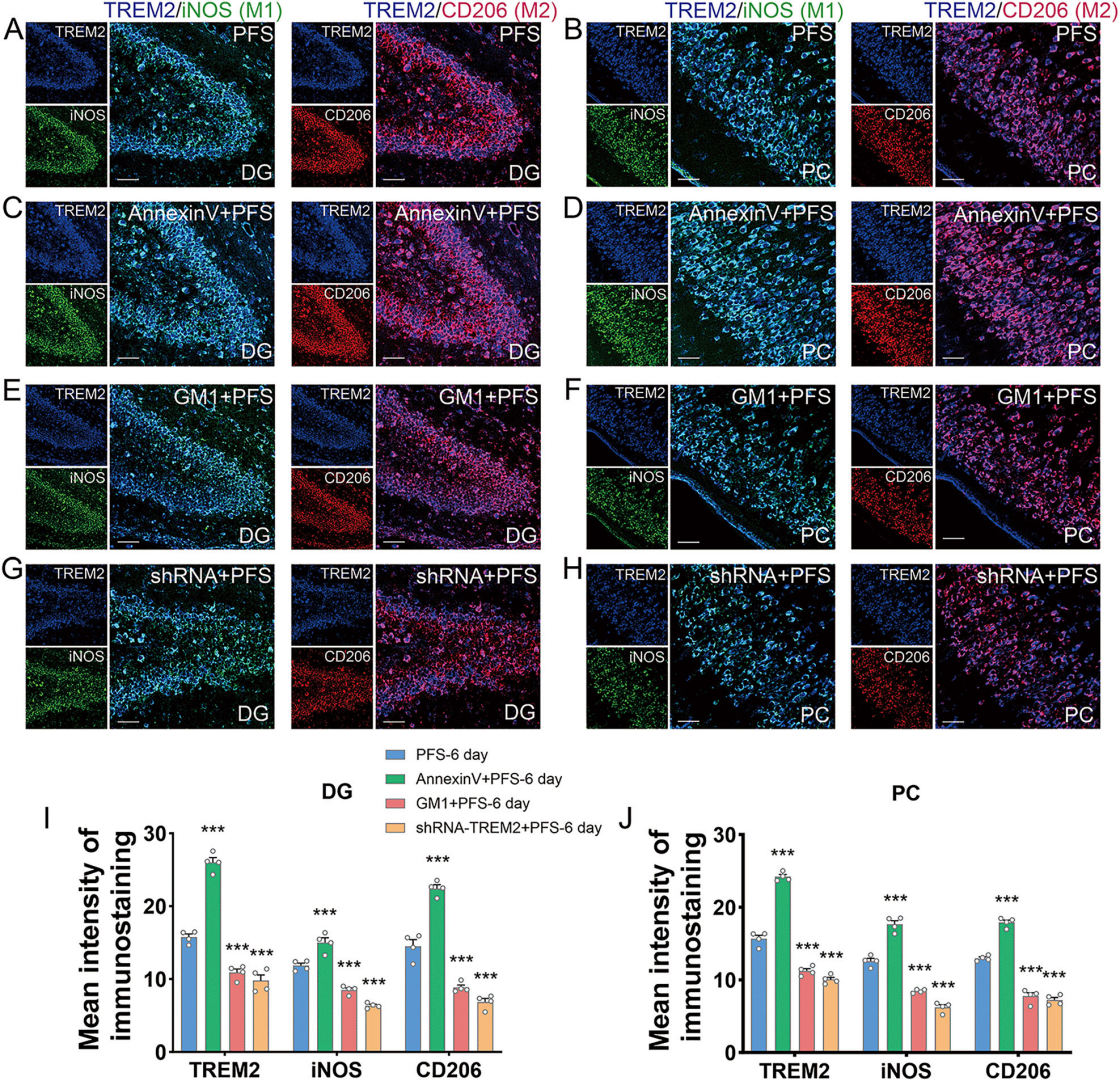
The expression changes of TREM2 in M1 and M2 microglia after drug intervention. **A**–**D** The immunohistochemical images of the changed levels of TREM2 (blue marker) and M1 type (iNOS, green), and M2 type (CD206, red) at 6 days after AnnexinV intervention in prolonged FS. **E**–**J** The immunohistochemical results of the changed levels of TREM2 (blue marker) and M1 type (iNOS, green), and M2 type (CD206, red) at 6 days after GM1 and shRNA intervention in prolonged FS. Scale bar = 50 μm, *n* = 4/group. Mean ± SEM were presented. ***p < 0.001 vs. control group (one-way ANOVA).

After GM1 and shRNA intervention, the level of the microglial cell marker TMEM119 significantly decreased (Fig. 3A, B, E–J). Furthermore, the co-localization of iNOS (Fig. 3A, B, E–J) and CD206 (Fig. 3A, B, E–J) was significantly decreased. Contrastingly, after annexin V intervention, the level of the microglial marker TMEM119 significantly increased (e.g., DG, *p* < 0.001; PC, *p* < 0.001; Fig. 3A–D, I, J), with upregulated levels of iNOS and CD206 in the hippocampus and cortical regions were significantly upregulated (Fig. 3A–D, I, J). Moreover, GM1 and shRNA intervention could significantly inhibit the expression of TREM2 in both types of microglia, while annexin V intervention significantly increased its level (Fig. 4A–J).

### Effects of TREM2 knockdown on the development of epilepsy induced by prolonged FS

**Fig. 5:**
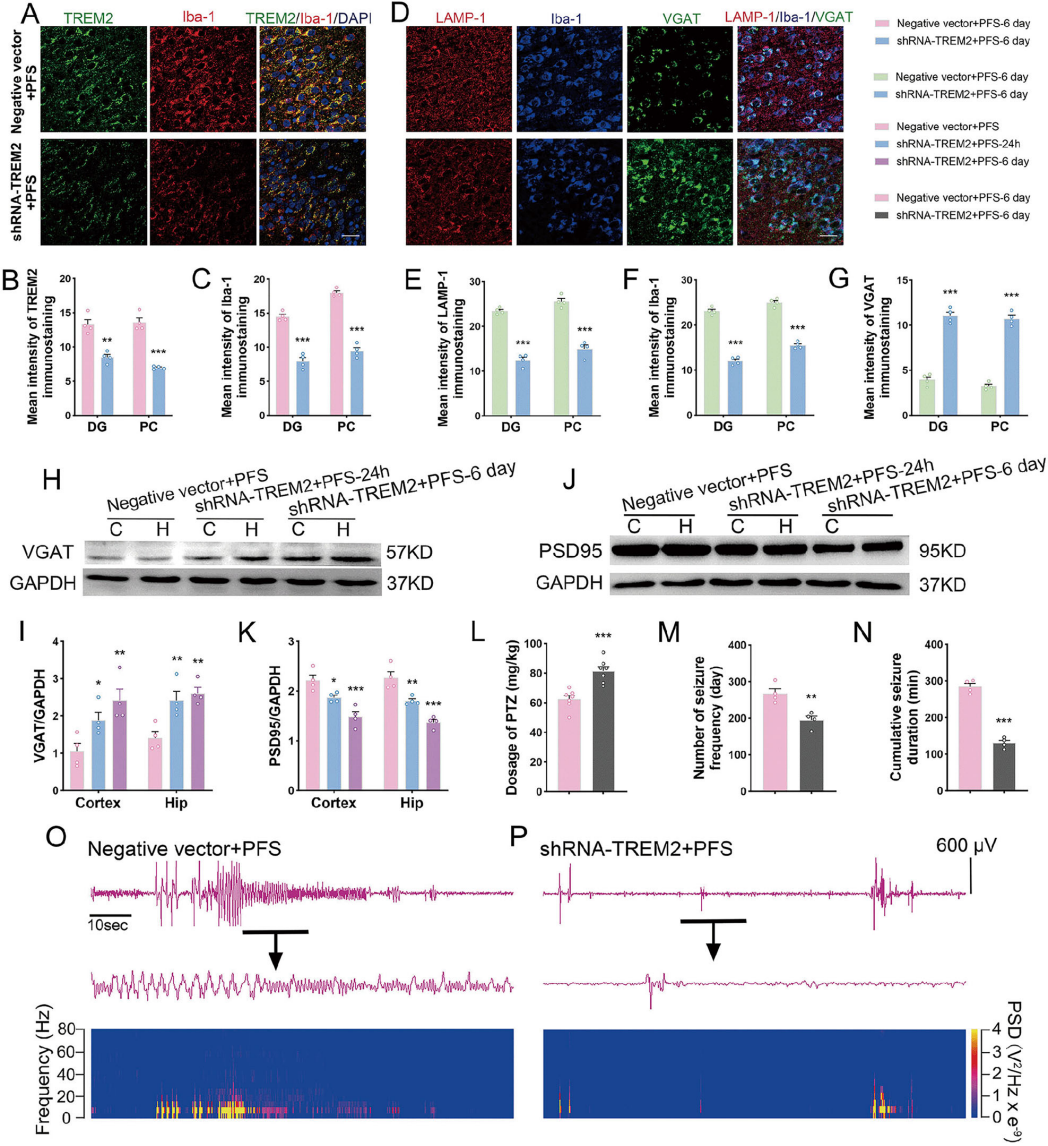
The changed levels of TREM2, microglial phagocytosis and epileptic seizures after shRNA intervention in prolonged FS rats. **A**–**C** The immunohistochemical results of the changes of TREM2 (green) and Iba-1 (red). Scale bar = 25 μm, *n* = 4/group, blue indicating DAPI staining. **D**–**G** The immunohistochemical results of LAMP-1 (red), Iba-1 (blue), and VGAT (green) after shRNA intervention (scale bar = 25 μm, *n* = 4/group). Gray bands and standardized gray values of VGAT (**H**, **I**) and PSD95 (**J**, **K**) relative to GAPDH (*n* = 4/group, one-way ANOVA). **L** Threshold dose of PTZ (*n* = 7/group). **M**, **N** Number of seizure frequency and cumulative seizure duration (*n* = 4/group). **O**, **P** The representative EEG and PSD analysis spectra of the animals. Mean ± SEM were presented. *p < 0.05, **p < 0.01, ***p < 0.001 vs. control group (unpaired *t*-tests).

Immunofluorescence staining results confirmed decreased levels of TREM2 in the hippocampus and cortex after shRNA intervention (Fig. 5A, B) as well as reduced Iba-1 (Fig. 5A, C, D, F) and lysosomal LAMP-1 (Fig. 5D, E and Supplementary Figs. 2, 6, 7), increased VGAT (Fig. 5D, G–I), and reduced PSD95 (Fig. 5J, K) levels.

The results of the behavioral experiments showed that the threshold dose of PTZ after shRNA intervention was significantly higher than that in the controls (*p* < 0.001, Fig. 5L), and that epileptic susceptibility was weakened. The EEG and PSD results showed that the frequency and duration of epileptic seizures in the shRNA intervention group were significantly lower than those in the control group (Fig. 5M–P). Thus, the ability of microglia to phagocytose inhibitory synapses, as well as the epileptic susceptibility and severity of epileptic seizures, were significantly attenuated after specific TREM2 knockdown using shRNA interference.

### Effects of reducing TREM2 expression by drug intervention on microglial synaptic phagocytosis and epileptic formation induced by prolonged FS

**Fig. 6:**
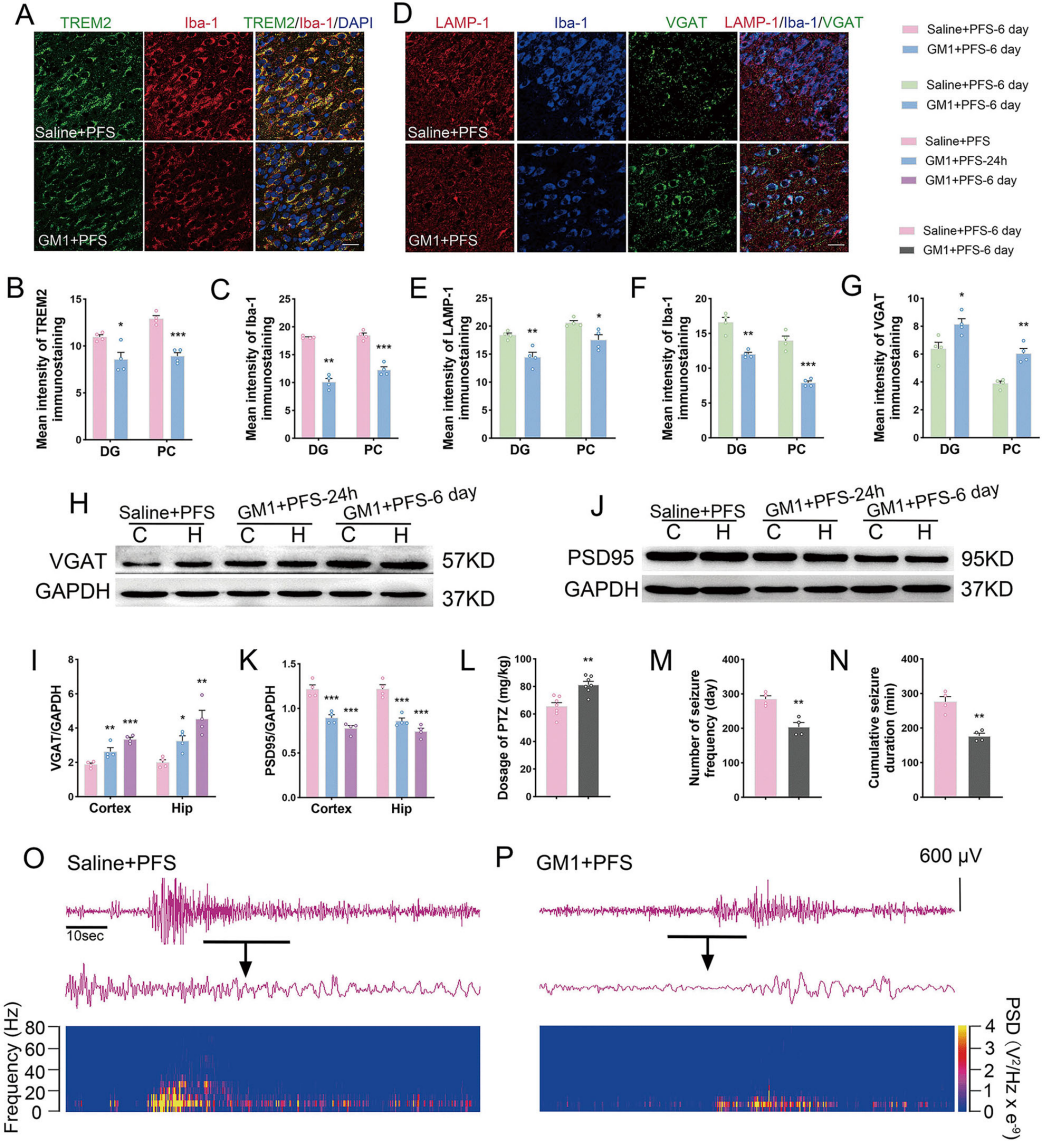
The changed levels of TREM2, microglial phagocytosis and epileptic seizures after GM1 intervention in prolonged FS rats. **A**–**C** Immunohistochemical results of TREM2 (green) and Iba-1 (red). **D**–**G** Immunohistochemical images and analysis of LAMP-1 (red), Iba-1 (blue), and VGAT (green). Scale bar = 25 μm, *n* = 4/group. Gray-scale strips and their standardized gray-scale values of VGAT (**H**, **I**) and PSD95 (**J**, **K**) relative to GAPDH (*n* = 4/group, one-way ANOVA). **L** Threshold dose of PTZ (*n* = 7/group). **M**, **N** Number of seizure frequency and cumulative seizure duration (*n* = 4/group). **O**, **P** The representative EEG and PSD analysis spectra of the rats. Mean ± SEM were presented. *p < 0.05, **p < 0.01, ***p < 0.001 vs. control group (unpaired *t*-tests).

As CD33 is considered a negative regulator of TREM2 [28], we administered GM1, a specific agonist of CD33, to assess the effects of changes in TREM2 levels on the phagocytosis of inhibitory synapses by microglia. GM1 administration reduced the levels of TREM2 (e.g. DG: *p* = 0.021, PC: *p* < 0.001, Fig. 6A, B), Iba-1 (Fig. 6A, C, D, F), and LAMP-1 (Fig. 6D, E) and increased the levels of VGAT (Fig. 6D, G–I and Supplementary Figs. 3, 6, 7). Western blotting revealed reduced levels of PSD95 (Fig. 6J, K).

Compared with the control group, the levels of p-DAP12 increased after prolonged FS stimulation (cortex, 6 days, *p* = 0.003; hippocampus, 6 days, *p* = 0.019; Supplementary Fig. 4A, C). After GM1 intervention, the upward trend of p-DAP12 in the prolonged FS model group was significantly reversed (Supplementary Fig. 4E, G). No significant differences in total DAP12 levels were found after prolonged FS and GM1 intervention (Supplementary Fig. 4B, D, F, H).

The epileptic susceptibility analysis confirmed that a higher dose of PTZ was required to induce generalized epileptic seizures after GM1 intervention (*p* = 0.001, Fig. 6L). The frequency and duration of epileptic seizures were reduced in animals treated with GM1 (Fig. 6M–P). These results indicate that GM1 may inhibit the ability of microglia to engulf inhibitory synapses by reducing TREM2 levels, thereby reducing epileptic susceptibility and seizures.

### Effects of blocking PtdSer to prevent TREM2 recognition on the development of epilepsy

**Fig. 7:**
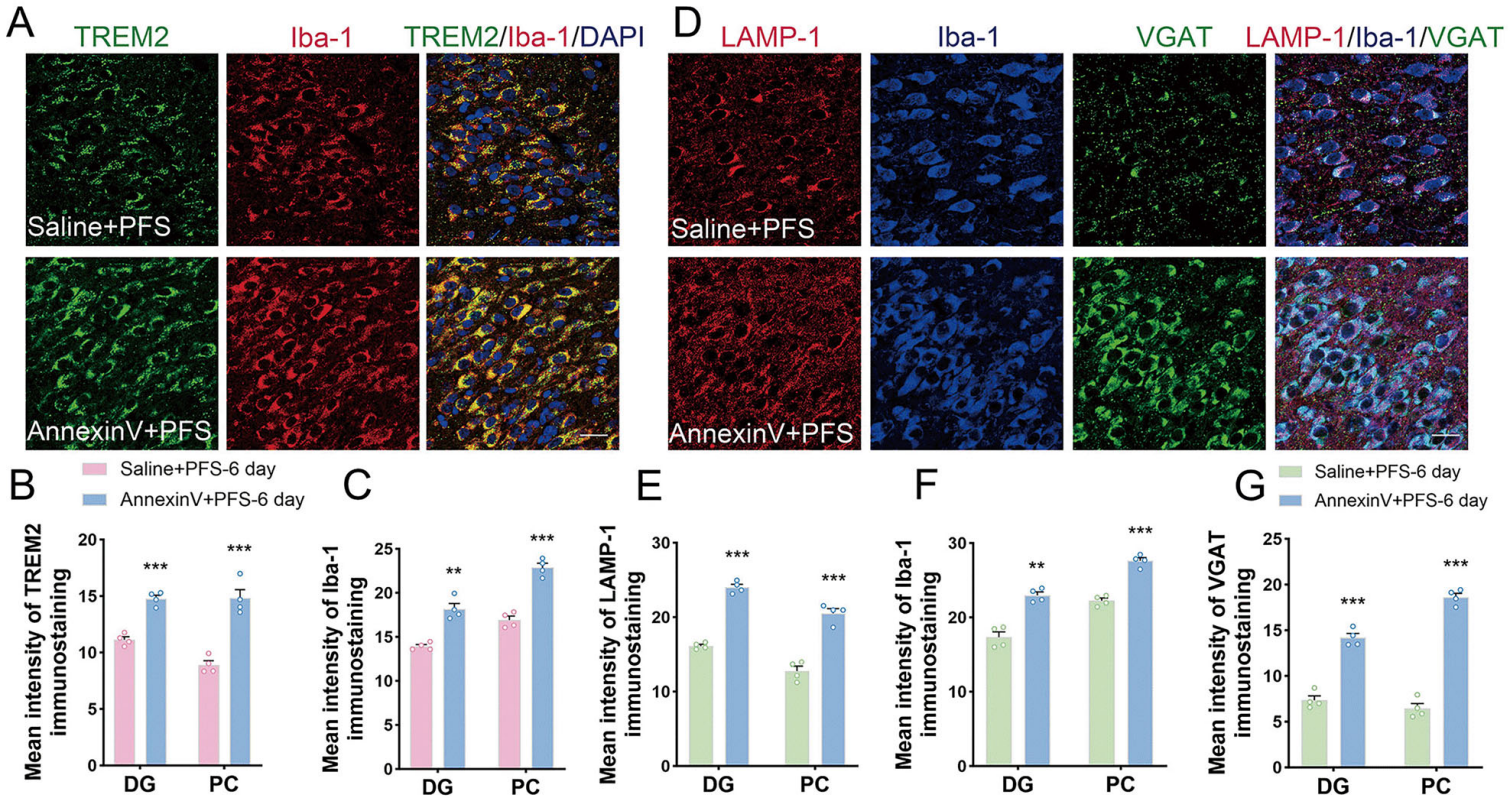
The increased levels of TREM2 and aggravated microglial phagocytosis after annexin V administration. **A**–**C** The immunohistochemical results of TREM2 (green) and Iba-1 (red). Scale = 25 μm, *n* = 4/group. Blue, DAPI. **D**–**G** The immunohistochemical results of LAMP-1 (red), Iba-1 (blue), and VGAT (green). Scale = 25 μm, *n* = 4/group. Mean ± SEM were presented. **p < 0.01, ***p < 0.001 vs. control group (unpaired *t*-tests).

**Fig. 8:**
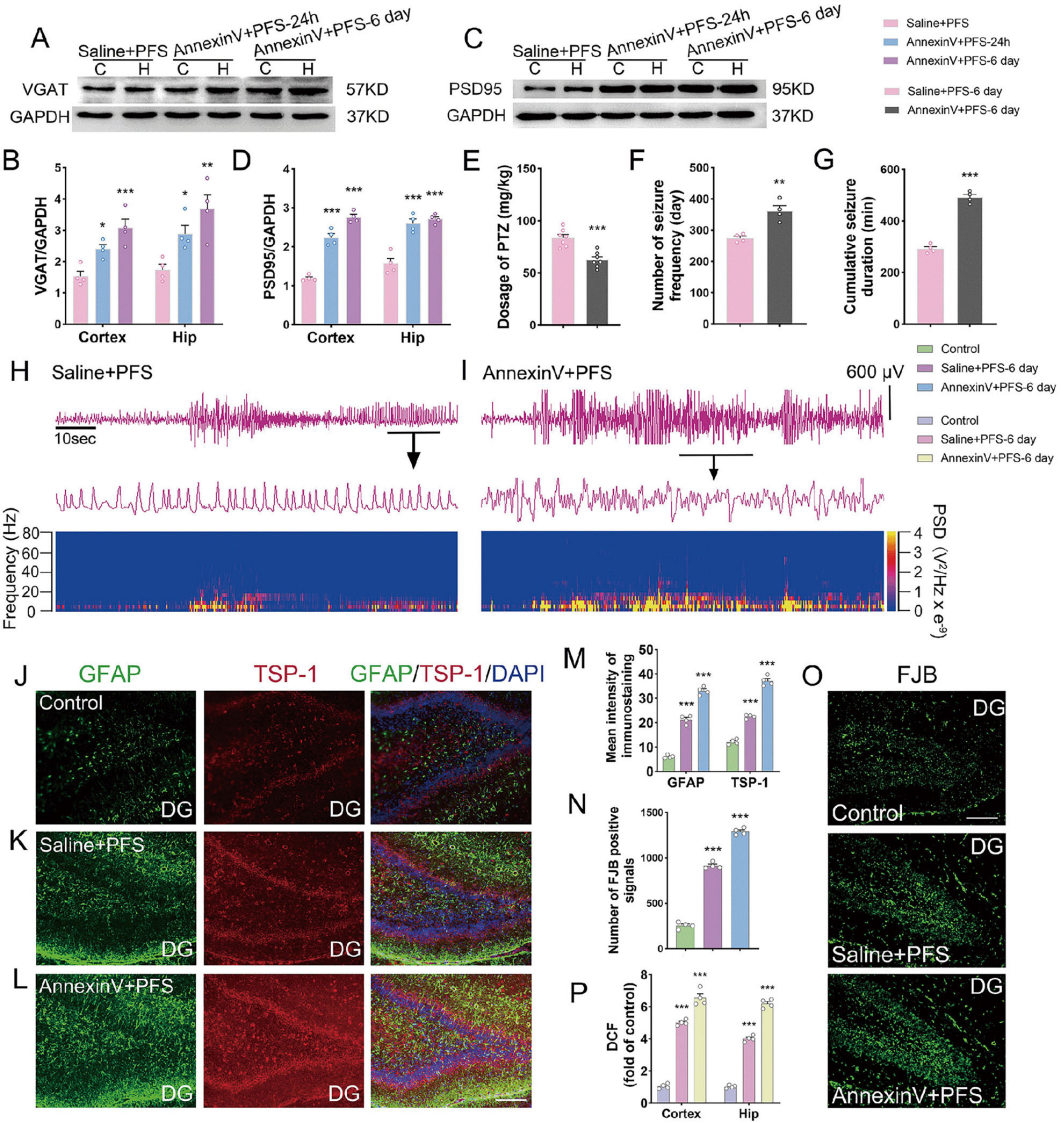
Changed synaptic levels, aggravated epileptic susceptibility, and epileptic seizures after annexin V administration. Gray bands and standardized gray value of VGAT (**A**, **B**) and PSD95 (**C**, **D**) relative to GAPDH (*n* = 4/group, One-way ANOVA). **E** Threshold dose of PTZ (*n* = 7/group). **F**, **G** Number of seizure frequency and cumulative seizure duration (*n* = 4/group). **H**, **I** the representative EEG and PSD analysis spectrum of the animals. **J**–**M** The immunohistochemical results of GFAP (green) and TSP-1 (red). Scale = 100 μm, *n* = 4/group. Blue, DAPI. **N**–**P** Fluoro-Jade B staining and oxidative stress results. Scale = 40 μm. Mean ± SEM were presented. *p < 0.05, **p < 0.01, ***p < 0.001 vs. control group (unpaired *t*-tests).

Annexin V was used to prevent TREM2 from recognizing PtdSer [29]. After annexin V intervention, the fluorescence intensity of the M1-type microglial marker iNOS and M2-type marker CD206 increased (*p* < 0.001, Fig. 3A–D, I, J). These results suggest that the M1/M2 polarization process of microglia was aggravated after annexin V treatment. Moreover, increased TREM2 levels were observed in both M1 and M2 microglia (Fig. 4A–D, I, J). Increased levels of TREM2 after annexin V administration (Fig. 7A, B) were accompanied by increased levels of Iba-1 (Fig. 7A, C, D, F), lysosomal LAMP-1 (Fig. 7D, E and Supplementary Figs. 5–7), VGAT (Figs. 7D, G and 8A, B), and PSD95 (Fig. 8C, D).

The dose of PTZ required to induce generalized epileptic seizures was significantly lower after annexin V intervention than in the controls (*p* < 0.001, Fig. 8E), and that susceptibility to epilepsy was enhanced. Moreover, EEG and PSD analysis spectra showed that epileptic seizures significantly worsened after annexin V intervention (increased seizure frequency and duration, Fig. 8F–I). These results suggest that when TREM2 fails to recognize synaptic PtdSer, the phagocytosis of inhibitory synapses by microglia decreases, while epileptic susceptibility and seizures increase. These results further confirm the crucial role of TREM2 in the development of epilepsy.

Further analysis was performed to explain the potential mechanism of aggravated epileptic seizures after annexin V intervention. The results revealed that the fluorescence intensity of GFAP (an astrocyte marker) in the annexin V intervention group was significantly higher, with increased levels of TSP-1 synchronously (*p* < 0.001, Fig. 8J–M). This indicated that annexin V intervention promoted the activation of astrocytes and upregulated TSP-1 secretion in prolonged FS rats. Additionally, neuronal injury (assessed by FJB-positive signals, Fig. 8N, O) and increased ROS levels (Fig. 8P) were aggravated. These results suggest that annexin V blocking the PtdSer site can accelerate oxidative stress damage, astrocyte activation, and TSP-1 secretion, although inhibitory synapses “escaped” phagocytosis.

## Discussion

FS is the most common seizure disorder in children and can have potentially serious consequences [30]. Besides obvious tonic-clonic seizures [30], FS can have long-term effects, including neuronal death, impaired synaptic plasticity, and spontaneous seizures [31, 32]. Our results showed that prolonged FS stimulation increased susceptibility to seizures and promoted epileptogenesis. During this process, TREM2-mediated microglial activation plays a critical role in phagocytizing inhibitory synapses. Furthermore, the inhibition or knockdown of TREM2 attenuates the targeting of inhibitory synapses by microglial phagocytosis and reduces epileptic susceptibility and epileptic seizures.

During the early stages of brain development in young rats, microglia present an amoeboid morphology characterized by large round cell bodies and a few short protrusions [33], as observed in the brains of rats in the prolonged FS group. This differs from mature microglia in adult rats, which have long, slender processes and smaller cell bodies [33]. This is because microglia are responsible for clearing most excess synapses to optimize the neural network during development. Amoeboid microglia recognize and phagocytose inefficient synapses through TREM2, phagocytose many naturally apoptotic neurons during development [34], and secrete growth factors that promote survival and integration of new neurons, thereby inhibiting excessive inflammation and preventing excessive pruning and neural damage [35]. However, in models of transient focal cerebral ischemia, microglial cell activation mainly gives rise to either M1 or M2 type microglia, which essentially represent a dynamic, initially pro-inflammatory (M1) or anti-inflammatory (M2) state [36]. In the initial stages of FS, M1 type microglia release pro-inflammatory factors and simultaneously generate large amounts of apoptotic debris [37]. However, in the FS state, activated microglia consist of both M1 and M2 types [38]. Phagocytic action is the core function of M2 microglia, which reduces pro-inflammatory factor levels, promotes phagocytosis, clears apoptotic debris, and prevents secondary inflammation [37]. TREM2 knockdown reduces the phagocytic ability of microglia [39]. Our results, in accordance with those of previous studies, indicate that TREM2 expression increases in M2 type microglia and that reducing TREM2 levels results in attenuated microglial activation and phagocytosis. Interestingly, TREM2 expression also increased in M1 type microglia, although the role and mechanism of action of TREM2 in these microglia require further research.

Regulation of microglial polarization is a classic intervention [40]. When microglia polarize into the pro-inflammatory phenotype (M1 type), they release large amounts of pro-inflammatory factors, amplify the neuroinflammatory response, and induce neuronal apoptosis and tissue damage [41]. By inhibiting the polarization of microglia toward the M1 phenotype, the release of pro-inflammatory factors can be significantly reduced and the inflammatory storm can be alleviated [42]. The transformation of pro-inflammatory M1-type microglia into an anti-inflammatory repair-type M2 phenotype may effectively alleviate excessive neuroinflammation in the brain, reduce neuronal damage, and improve synaptic plasticity [40, 42].

Both CD33 (also known as Siglec-3) and TREM2 are immune regulatory receptors on the surface of myeloid cells; however, they have opposing functions and are mutually inhibitory [43, 44]. As an upstream molecule of TREM2, CD33 regulates both TREM2 expression and the inflammatory response and phagocytic function of microglia by inhibiting the TREM2 signaling pathway [28]. TREM2 binds to the adapter protein DAP12, and the immunoreceptor tyrosine-based activation motif (ITAM) of DAP12 is phosphorylated after ligand binding to recruit Syk kinase and activate downstream pathways [45]. The intracellular segment of CD33 contains an immunoreceptor tyrosine-based inhibition motif (ITIM), which is phosphorylated and then recruits the SHP-1/SHP-2 phosphatases [46], accordingly, the ITAM motif is directly dephosphorylated in the TREM2-DAP12 complex, blocking the recruitment of Syk kinase and thereby inhibiting the phagocytic function of microglia regulated by TREM2 [47]. The effects of regulating the expression of CD33 and influencing the phagocytic function of microglia can be eliminated by knocking out TREM2 [28]. Our results showed that the expression of TREM2 increased and that of CD33 decreased in the brains of rats with prolonged FS and that the ability of microglia to phagocytose inhibitory synapses increased accordingly. Moreover, the activation of CD33 led to reduced TREM2 levels, attenuated microglial phagocytosis of inhibitory synapses, and decreased epileptic susceptibility. TREM2 knockdown yielded similar results. Thus, decreasing TREM2 levels directly or indirectly by activating CD33 may be a potential method for preventing prolonged FS from developing into epilepsy.

PtdSer is a major phospholipid component of the inner layer of the cell membrane, and its externalization is an important biological signal in several critical processes such as apoptosis, synaptic pruning, and immune regulation [48]. In normal cell membranes, PtdSer is distributed almost entirely (>95%) in the inner cytoplasmic side of the plasma membrane [49]. Under conditions of nerve injury or oxidative stress, reactive oxygen species (ROS) disrupt membrane asymmetry, leading to the translocation of PtdSer from the inner to outer membranes of synapses [29, 50]. Oxidative stress is a major feature of overexcitation [51]; additionally, excessive synaptic excitation (such as glutamate excitotoxicity) can lead to Ca^2+^ influx [52], activate Ca^2+^-dependent phosphatidylserine scramblases, and promote the exposure of PtdSer [53]. The PtdSer protrusion acts as a “eat-me” signal, subsequently, TREM2 recognizes inverted PtdSer and activates microglia, causing them to engulf inhibitory synapses in large quantities [29], thereby disrupting the balance between excitation and inhibition in the brain. Since both FS and epilepsy are closely related to overexcitation, it is reasonable to speculate that inverted PtdSer-triggered TREM2-mediated microglial phagocytosis contributes to FS-induced epileptogenesis.

We blocked PtdSer sites to investigate the changes in microglial phagocytosis. Interestingly, aggravation of microglial activation and epileptic seizures was observed in response to annexin V treatment, although the number of inhibitory synapses significantly increased. Annexin V is a Ca^2+^-dependent phospholipid-binding protein [54] with high sensitivity and specificity for binding and blocking PtdSer to prevent TREM2 from recognizing [29]. Our results showed that blocking the PtdSer site promotes microglial activation in the prolonged FS group. Notably, when the TREM2-PtdSer pathway is blocked, microglia may be activated via other pathways. Though inhibitory synapses were “escaped” from being phagocytosed, the accumulation of damaged substances cannot be effectively cleared, may release damage-related factor patterns (DAMPs) [55], continuously activating the type M1 microglia to secrete large amounts of pro-inflammatory factors [56, 57], which play vital roles in promoting the epileptic development via multiple pathway [58, 59]. For example, these pro-inflammatory factors can induce astrocyte activation [58]. Activated astrocytes can promote the proliferation of excitatory synapses and vascular damage during epileptic development via TSP-1 mediation [59–61]. Our results confirmed that after annexin V intervention, increased TSP-1 levels and astrocyte activation were further aggravated, accompanied by higher levels of excitatory synapses. These results indicate that the aggravated levels of astrocyte activation and TSP-1 increase may contribute to the proliferation of excitatory synapses, further aggravating the excitation/inhibition imbalance.

Moreover, pro-inflammatory factors released from activated M1 microglia may induce mitochondrial damage and generate reactive oxygen species (ROS) [62]. Excessive accumulation of ROS plays a vital role in epileptic development by causing oxidative stress damage to mitochondria and neurons [63], eventually prompting presynaptic neurons to release excitatory amino acids such as glutamate, thereby triggering epileptic seizures [64]. Moreover, reducing ROS levels results in reduced release of excitatory amino acids and relieves seizures in epilepsy [65–67]. Our results confirmed aggravated oxidative stress and neuronal injury resulting from the annexin V intervention. Consequently, although inhibitory synapses “escape” phagocytosis by activated M2 microglia, prolonged FS-induced epileptic seizures may be aggravated by the contribution of M1 microglia via multiple mechanisms such as pro-inflammatory factor release and interaction with astrocytes.

Interestingly, we found that increased TREM2 levels was accompanied by a reduction in inhibitory synapses and an increase in excitatory synapses after prolonged FS. This may be due to the complexity of the various contributors participating in the phagocytic targeting of synapses. Complement system proteins such as C1q and C3 also act as “eat-me” signals for microglial phagocytosis, and the recognition of complement markers (such as C3) by microglia and preferential phagocytosis of inhibitory synapses marked by C3 is enhanced upon activation of TREM2 [68]. During the pathological process, inhibitory synapses are prone to being marked, and phagocytosed because of their reduced activity [68]. However, excitatory synapses (glutamatergic) may express CD47 (a “don’t eat me” signal) or lack complement markers [69]. Moreover, microglia may secrete brain-derived neurotrophic factor (BDNF) and enhance synaptic strength via tropomyosin receptor kinase B (TrkB) receptors [35]. Additionally, activated astrocytes may participate in the proliferation of excitatory synapses during epileptogenesis through TSP-1/TGF-β1 pathway [27, 70, 71]. Moreover, glycosylphosphatidylinositol-anchored proteoglycan 4/6 (Gpc4/6) secreted by astrocytes is an important factor in inducing the formation of functional excitatory synapses, specifically promoting membrane transport and synaptic site aggregation of AMPA (α-amino-3-hydroxy-5-methyl-4-isoxazolepropionic acid) receptors in neurons, promoting the maturation and increasing the density of functional excitatory synapses [71]. Consequently, in addition to TREM2-regulated phagocytosis of inhibitory synapses by microglia, excitatory synapses escape phagocytosis, and excitatory synaptogenesis may also have a combined effect in prolonged FS-induced epilepsy.

## Conclusions

FS is a major cause of epilepsy and there are currently no effective drugs to prevent its progression to epilepsy. Our results suggest that TREM2-depended microglial activation contributes to the phagocytosis of inhibitory synapses, and that reducing TREM2 expression may be a potential therapeutic approach to prevent prolonged FS from developing into epilepsy. A limitation of the present study is that the shRNA intervention was administered before FS. The post-treatment effects of TREM2 intervention will be investigated in future studies.

## Methods

### Animals

Male and female Sprague-Dawley rats aged 2 months (Pengyue Experimental Animal Center, No. SCXK 2022-0006, Jinan, China) were housed in transparent ventilated cages (two females and one male/cage), whereas pregnant rats were housed in a single cage. The experimental animals were provided adequate food and water, and efforts were made to minimize the number of animals and their suffering during the experiments. All experiments were conducted in accordance with the ethical guidelines of the Committee on Animal Experimentation of Binzhou Medical College (approval no. 2023003) and the National Institutes of Health Guidelines on the Care and Use of Laboratory Animals (National Institutes of Health Publication No. 80-23, 1996 revision).

Based on the experimental setup, there were four arms in this study. In the first arm, pups from the same litter were randomly divided into prolonged FS and control groups. In the second arm, pups from the same litter were divided into the shRNA + prolonged FS and negative vector + prolonged FS groups. Similarly, pups were treated with monosialoganglioside 1 (GM1) or annexin V and controls were treated with saline. A total of 300 Sprague-Dawley rat pups were used in this study: 265 pups were used to model prolonged FS, and 257 rat pups were successfully modeled. Eight pups that died (4 pups, mortality rate of 1.51%) from serious seizures or did not experience seizures for more than 55 min during the modeling process were excluded from further analysis. The investigator was blinded to the group collocation during the experiment.

### Prolonged FS

The day of the pups’ birth was designated postnatal day 0 (P0). Based on previous reports [72–74], P8–P9 rats from the same litter were randomly divided into prolonged FS and control groups, and animals with body weight of 15–18 g were included. The pups in the prolonged FS group were placed in an incubator at a constant temperature (44 ± 0.5 °C) for FS management. Episodes of rats experiencing prolonged absent-mindedness or hind limb weakness and then walking in a “swimming” position were judged to be seizures [75]. After recording their body temperatures, the pups were removed from the incubator, kept at 25 °C for 2 min, and placed in the incubator again to induce a second seizure. After 10 min of seizures, the pups were removed and moved to 25 °C for 2 min, followed by the third and fourth seizure treatments. During the modeling process, the total seizure time for each rat was no less than 30 min [25]. Pups that died or did not experience seizures for >55 min were excluded from further analysis. After modeling, the pups were placed back in their cages.

### Drug intervention

The expression of TREM2 is inhibited by CD33 activation. GM1, which acts as a CD33 agonist [28], was administered daily (intraperitoneally [i.p.], 10 mg/ml, 20 mg/kg; Qilu Pharmaceutical Co., Ltd.) 2 h before FS modeling. Annexin V was used to block the membrane structure at the eversion site of phosphatidylserine (PtdSer), preventing its recognition by TREM2 [76]. Annexin V (i.p., once every 4 days, 100 ug/kg, 10448-HNAE, Sino Biological) was administrated 2 h before FS modeling. Animals in the control group were administered saline.

### shRNA intervention

Three days before FS modeling, the rats were fixed on a stereotaxic device (Anhui Zheng Hua Biological Instrument Equipment Co., Ltd., China) after 20 s of exposure to ether inhalation anesthesia (CAS, 60-29-7, Guoyao, China). To evaluate TREM2 expression, an encoded lentivirus (3 × 10^7^ TU/kg, LVCON313; Jikai Gene, China) targeting TREM2 was injected into the lateral ventricle (anteroposterior [AP]: 2 mm; mediolateral [ML]: 1.5 mm; dorsoventral [DV], 3 mm). The oligonucleotide sequence was 5’-CTGCGTTCTCCTGAGCAAGTT-3’. The control sequence of negative vector was 5’-TTCTCCGAACGTGTCACGT-3’.

### Western blot analysis

After anesthesia (sodium pentobarbital, 50 mg/kg, i.p.; CAS, 57-33-0, Xiya Reagent, China), the brains were separated on ice at 24 h and 6 days after FS. Then RIPA lysate buffer (Beyotime, P0013B) and phenylmethanesulfonyl fluoride (Beyotime, ST506) was added, followed by centrifugation (4 °C for 20 min). The concentration of the supernatant was measured using a bicinchoninic acid protein assay kit (Beyotime, P0012). Based on these results, the concentration of the supernatant was balanced by adding RIPA lysate. Proteins were separated on a 10% sodium dodecyl sulfate-polyacrylamide gel (Beyotime, P0012AC) and transferred to polyvinylidene difluoride (PVDF) membranes (VGAT, 60 min; PSD95, 90 min; CD33, 75 min; phospho-DAP 12/DAP 12/GAPDH, 50 min), which were blocked using 5% skim milk for 2 h and then incubated with rabbit monoclonal antibody anti-PSD95 (1:1000; ab18258; Abcam), anti-VGAT (1:1000; ab42939; Abcam), anti-CD33 (1:1000; 77576S; Cell Signaling Technology), anti-Phospho-DAP12 (1:1000; 32308; Cell Signaling Technology), anti-DAP12 (1:1000; 12492; Cell Signaling Technology) or anti-GAPDH (1;1000; AB-P-R001; Goodhere) with slow shaking overnight at 4 °C. After incubation with horseradish peroxidase-conjugated IgG (1:3000; ZB-2301; Beijing Zhongshan) on an orbital shaker at 25 °C for 1.5 h, images were acquired using a biomolecular imager (Tanon 4600; Tanon, Shanghai, China). ImageJ software (version 1.49; National Institutes of Health, Bethesda, MD, USA) was used for further analysis. The gray values of all target films were normalized to those of GAPDH.

### Epileptic susceptibility assessment

Pentylenetetrazole (PTZ; 0.05 ml/min, 5 mg/ml; Sigma) was administered via tail vein injection to P14 rats until they developed grade 4–5 seizures (as judged using the Racine criteria) [26, 27]. At this age, the rats could walk upright and experienced significant systemic seizures after treatment with PTZ. The threshold dose was calculated as follows: PTZ threshold dose (mg/kg) = PTZ concentration (mg/ml) × infusion volume (ml)/rat body weight (kg) [77, 78].

### Immunohistochemistry

The rats were anesthetized with pentobarbital sodium (50 mg/kg, i.p.; CAS, 57-33-0, Xiya Reagent, China) at 24 h and 6 days after modeling. Thereafter, 40 ml of 0.9% normal saline was perfused through the systemic circulation until the liver turned white, followed by fixation with 40 ml 4% paraformaldehyde (PAF). Subsequently, the brains were fixed in PAF for 24 h and dehydrated in 15% and then 30% sucrose. Eventually, 11 µm sections were cut at −27 to −28 °C in a cryostat microtome (CM1860, Leica, Germany), washed with 0.01 M phosphate buffered saline (PBS) three times, treated with citric acid repair solution at 80 °C for 20 min (Solarbio, C1010, China), immersed in 1% Triton-100 (MeilunBio, MB2486) at 25 °C, and then shielded from light for 30 min. After blocking with 10% bovine serum protein (BSA) (MeilunBio, MB4219) at indoor temperature for 2 h, the slices were incubated in the primary antibodies—rabbit monoclonal antibody, anti-TREM2 (1:200; 91068S; Cell Signaling Technology), anti-thrombospondin-1 (TSP-1; 1:200; ab85762; Abcam), anti-LAMP1 (1:200; ab237307; Abcam); anti-IBA-1 (goat monoclonal antibody; 1:200; NB100-1028; NOVUSBIO); mouse monoclonal antibody, anti-VGAT (1:200; ab211534; Abcam), anti-GFAP (1:200; BM0055; BOSTER Biological Technology) at 4 °C for 24 h. This was followed by incubation with the corresponding secondary antibodies—anti-FITC (goat anti-rabbit IgG antibody; 1:200; A0562; goat anti-mouse IgG antibody; 1:200; A0568; Beyotime), anti-Dylight 405 (goat anti-rabbit IgG antibody; 1:200; A0605; Beyotime; rabbit anti-goat IgG antibody; 1:200; A23130; Abbkine), anti-Cy3 (donkey anti-goat IgG antibody; 1:200; A0502; Beyotime) at 25 °C for 1.5 h in the dark. After 4’,6-diamidino-2-phenylindole (DAPI; 1:3000; 50 ul/section; C1005; Beyotime) incubation at indoor temperature for 15 min in the dark, the brain sections were sealed with a cover glass (Solarbio, S2100), and images were acquired using a laser confocal microscope (LSM880, Zeiss, Germany). Fluorescence intensity was analyzed using ZEISS ZEN 3.11 software.

### Fluoro-Jade B (FJB) staining

FJB staining has been used to specifically label degenerated neurons and assess neuronal damage in the brain [79]. Brain slices from the prolonged FS model 6 days after modeling were dried at 37 °C for 30 min. They were then dehydrated in a 1% sodium hydroxide (NaOH) ethanol solution and 70% ethanol solution for 5 min each, followed by washing with ddH_2_O for 2 min. The slices were incubated in 0.06% potassium permanganate solution in the dark for 15 min, washed with ddH_2_O for 2 min to reduce background, immersed in 0.0004% FJB staining solution (FJB dissolved in 0.1% acetic acid solution), and incubated in the dark for 35 min. After washing with ddH_2_O, the slices were soaked in xylene for 2 min and dried in a fume hood. Images were captured using a fluorescence microscope (Olympus, Tokyo, Japan).

### Reactive oxygen species (ROS) detection

To assess oxidative stress levels after microglial activation, we measured the relative ROS content [63]. The rats were anesthetized, and the brain tissues were rapidly dissected. Under ice bath conditions, the hippocampus and cortex tissues were precisely separated, and 10 µL/mg of pre-cooled 0.01 M PBS was added, followed by cutting into small pieces and filtering through a 200-mesh cell sieve to collect the single-cell suspension. A total of 250 µL of the single-cell suspension was taken and mixed with 500 µL of 2’, 7’-dichlorodihydrofluorescein diacetate (DCFH-DA, S0033S, Beyotime, China, dilute with serum-free medium at a ratio of 1:1000 to obtain a concentration of 10 μmol/L) working solution and incubated in the dark at 37 °C for 40 min. The cell suspension was then centrifuged at 2000 rpm at 4 °C, and the supernatant was discarded. This centrifugation and washing step was repeated thrice. The fluorescence signal was detected using a multifunctional microplate reader (Synergy H1; BioTek Instruments, Inc., USA) at excitation and emission wavelengths of 488 and 525 nm, respectively. ROS levels were characterized by the relative fluorescence intensity.

### Electroencephalographic (EEG) recording and analysis

The EEGs of P14 rats were recorded after prolonged FS. After anesthesia with pentobarbital sodium, the rats were immobilized using a stereotaxic device (Anhui Zheng Hua Biological Instrument Equipment Co., Ltd., China). Stainless steel electrodes (0.5 mm tip uncoated; A.M. Stems, USA) were implanted in the left cortex [AP, −2.3 mm; ML, −2.1 mm; DV, −1.3 mm]. Seven days after surgery, EEGs were recorded for three consecutive days. The PowerLab system (1-50HZ, AD Instruments, Australia) was used to record and analyze the data.

### Statistical analyses

All data were presented as mean ± SEM values and were statistically analyzed using SPSS (version 25.0; IBM, USA). Based on the data obtained from our pre-experimental analyses, the sample size was estimated using balanced one-way analysis of variance (ANOVA). As the data conformed to the normal distribution and homogeneity of variance, one-way ANOVA was used to analyze one categorical independent variable between three or more groups, and an unpaired *t*-test was used to analyze the data of one categorical independent variable between two groups. Statistical significance was set at *p* < 0.05.

## Supplementary information

Supplementary Figures legends

Original gel bands of Figures 1

Original gel bands of Figures 2

Supplementary Figure 1

Supplementary Figure 2

Supplementary Figure 3

Supplementary Figure 4

Supplementary Figure 5

Supplementary Figure 6

Supplementary Figure 7

## Data Availability

The data and material in this study are available from the corresponding author on reasonable request.
