# Supplementary material for: TREM2-mediated microglial phagocytosis of inhibitory synapses contributes to prolonged FS-induced epileptogenesis
Source: Cell Death Discov. 2026 Apr 11;12:223. doi: 10.1038/s41420-026-03118-7 (PMC13184081; doi:10.1038/s41420-026-03118-7)
Supplement: Supplementary file 1 — Supplementary Figures legends [file 41420_2026_3118_MOESM1_ESM.docx]

**Supplementary Figure 1**

**
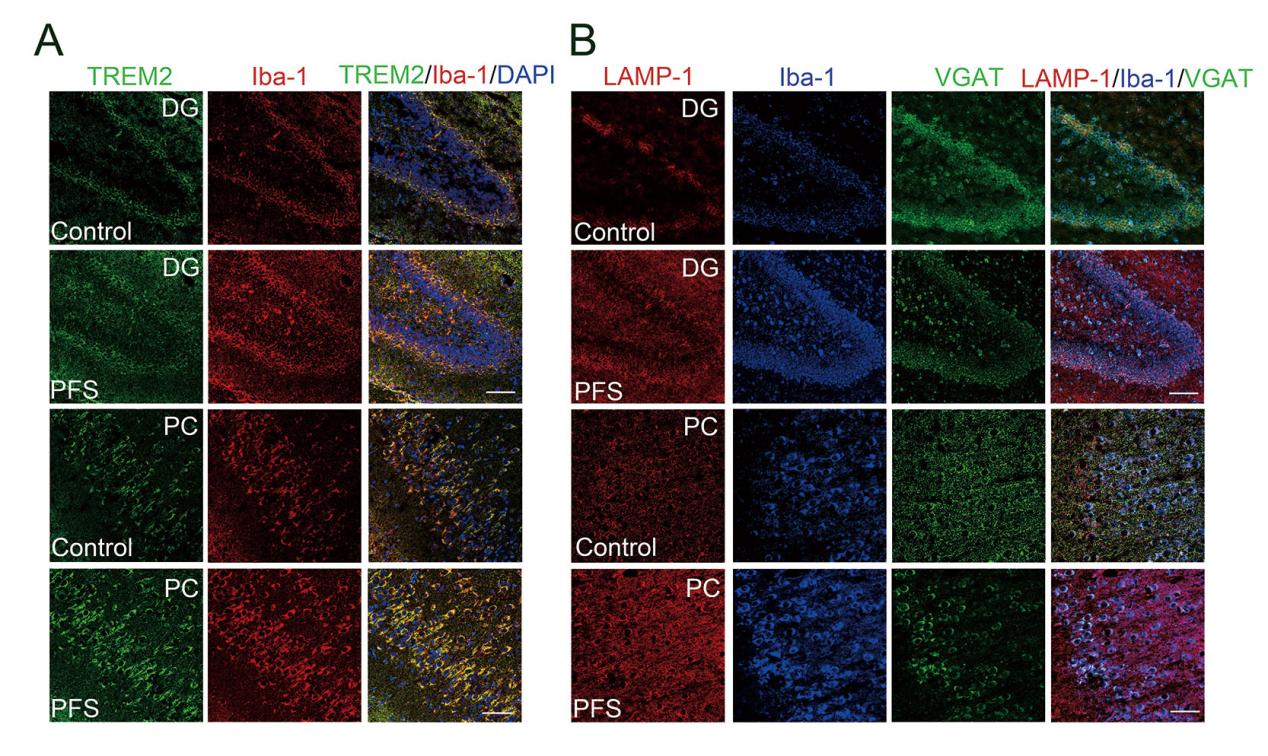
**

**Supplementary Figure 1 Increased TREM2 levels and microglial activation in the brains of animals with prolonged FS.**

**(A)** The immunohistochemical results of TREM2 (green) and Iba-1 (red). Blue, DAPI. **(B)** The immunohistochemical results of LAMP-1 (red), Iba-1 (blue), and VGAT (green). Scale = 50 μm.

**Supplementary Figure 2**

**
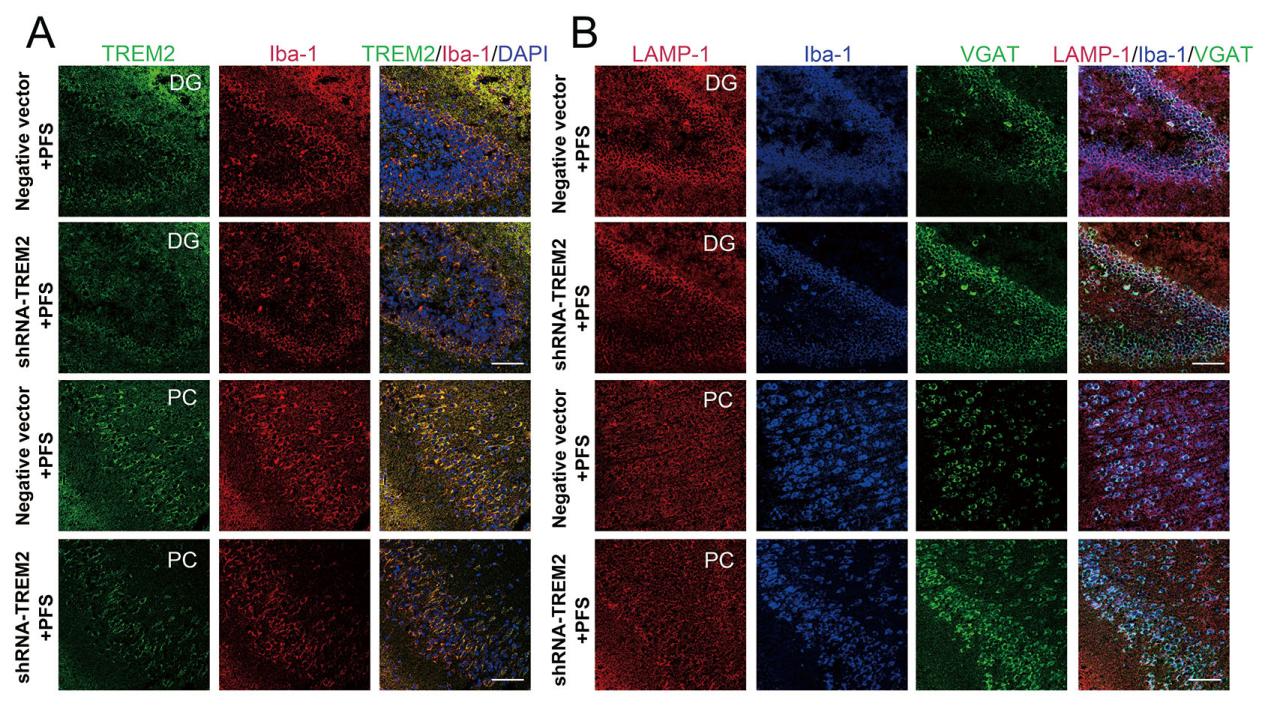
**

**Supplementary Figure 2 The changed levels of TREM2 and microglial phagocytosis after shRNA intervention.**

**(A)** The immunohistochemical results of TREM2 (green) and Iba-1 (red). Blue, DAPI. **(B)** The immunohistochemical results of LAMP-1 (red), Iba-1 (blue), and VGAT (green). Scale = 50 μm.

**Supplementary Figure 3**

**
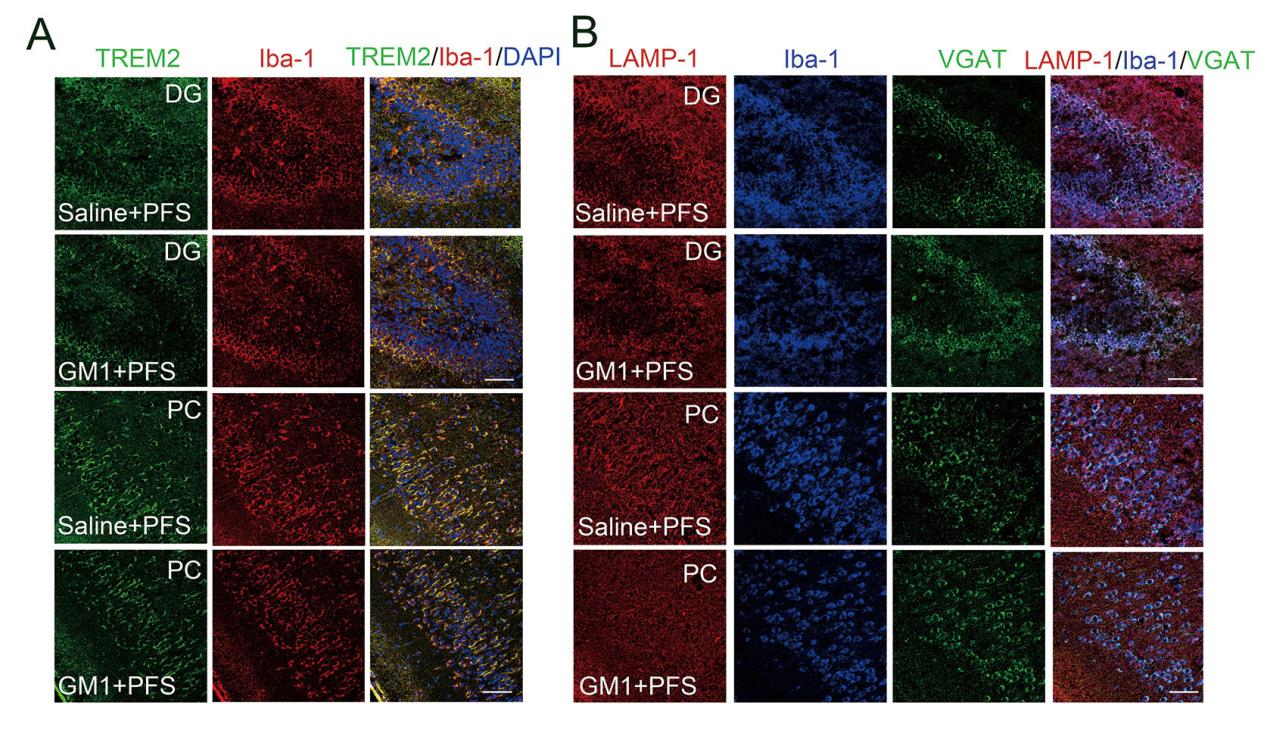
**

**Supplementary Figure 3 The changed levels of TREM2 and microglial phagocytosis after GM1 administration.**

**(A)** The immunohistochemical results of TREM2 (green) and Iba-1 (red). Blue, DAPI. **(B)** The immunohistochemical results of LAMP-1 (red), Iba-1 (blue), and VGAT (green). Scale = 50 μm.

**Supplementary Figure 4**

**
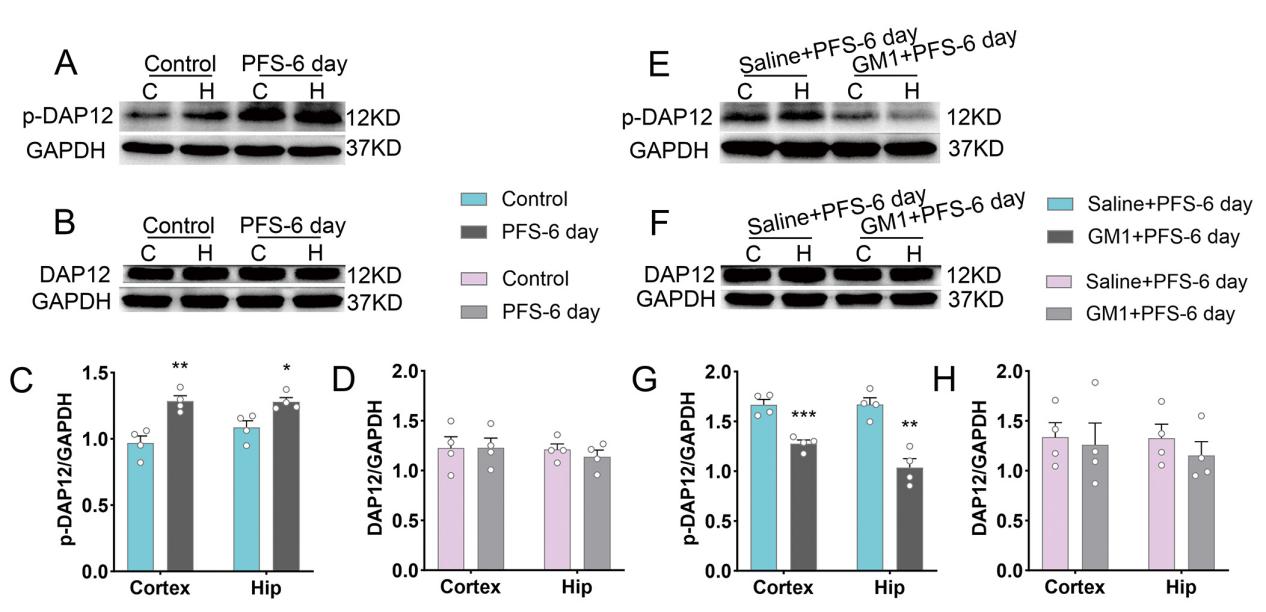
**

**Supplementary Figure 4 The changed levels of p-DAP12 and DAP12 in the hippocampus and cortex after GM1 intervention in prolonged FS rats.**

The gray-scale bands of p-DAP12, DAP12 **(A, B)** and analysis **(C, D)** at 6 days after prolonged FS (n = 4/group). The gray-scale bands of p-DAP12, DAP12 **(E, F)** and analysis **(G, H)** after GM1 intervention in prolonged FS (n = 4/group). Mean ± SEM were presented. *P < 0.05 **P < 0.01, ***P < 0.001 *vs* control group (unpaired t-tests)

**Supplementary Figure 5**

**
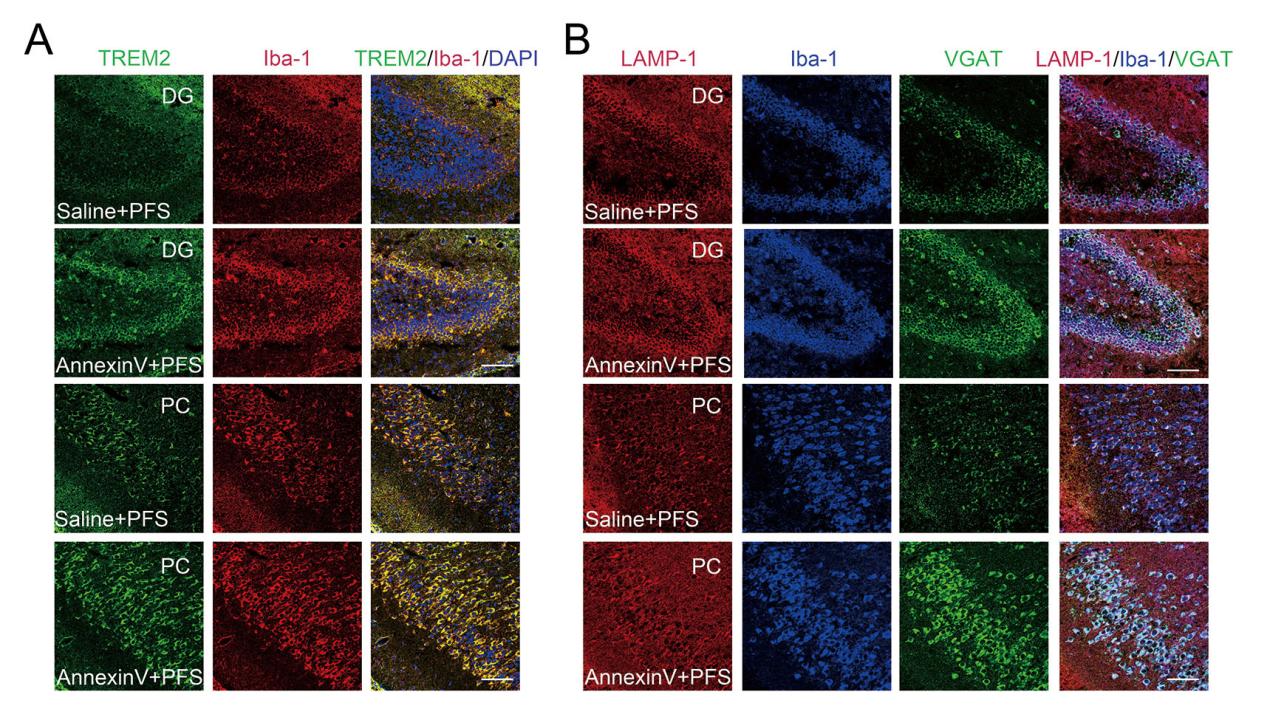
**

**Supplementary Figure 5 The changed levels of TREM2 and microglial phagocytosis after Annexin V administration.**

**(A)** The immunohistochemical results of TREM2 (green) and Iba-1 (red). Blue, DAPI. **(B)** The immunohistochemical results of LAMP-1 (red), Iba-1 (blue), and VGAT (green). Scale = 50 μm.

**Supplementary Figure 6**


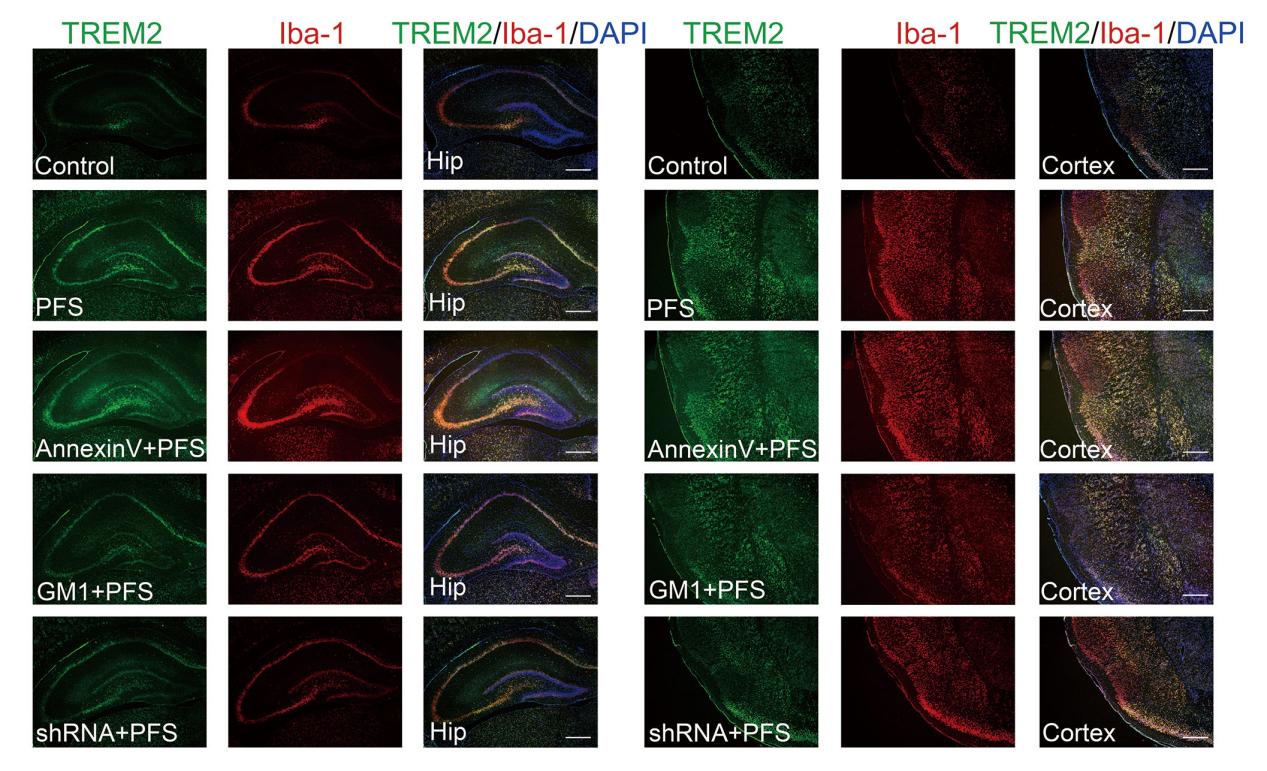


**Supplementary Figure 6 The changed levels of TREM2 and microglial phagocytosis in the hippocampus and cortex of rats.**

The immunohistochemical results of TREM2 (green) and Iba-1 (red) in the hippocampus and cortex. Scale = 200 μm, n = 4/group. Blue, DAPI.

**Supplementary Figure 7**

**
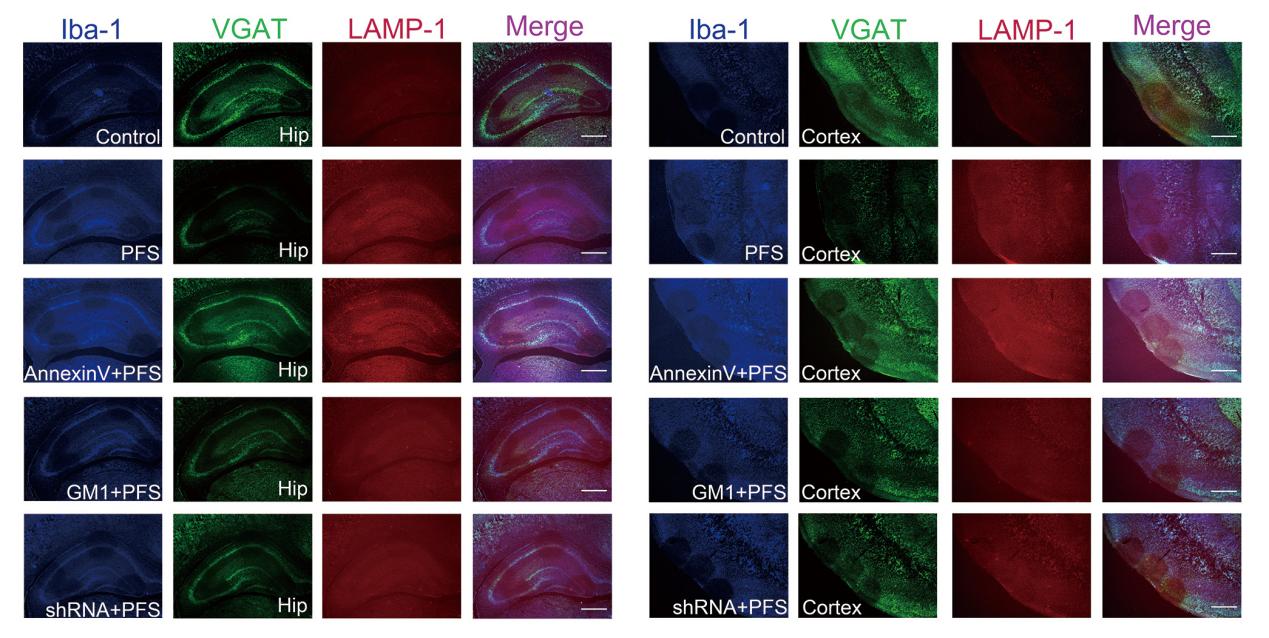
**

**Supplementary Figure 7 The changed levels of VGAT and microglial phagocytosis in the hippocampus and cortex.**

The immunohistochemical results of Iba-1 (blue), VGAT (green) and LAMP-1 (red) of hippocampus and cortex. Scale = 200 μm, n = 4/group.
