# Supplementary figures and images for: TREM2-mediated microglial phagocytosis of inhibitory synapses contributes to prolonged FS-induced epileptogenesis
Source: Cell Death Discov. 2026 Apr 11;12:223. doi: 10.1038/s41420-026-03118-7 (PMC13184081; doi:10.1038/s41420-026-03118-7)

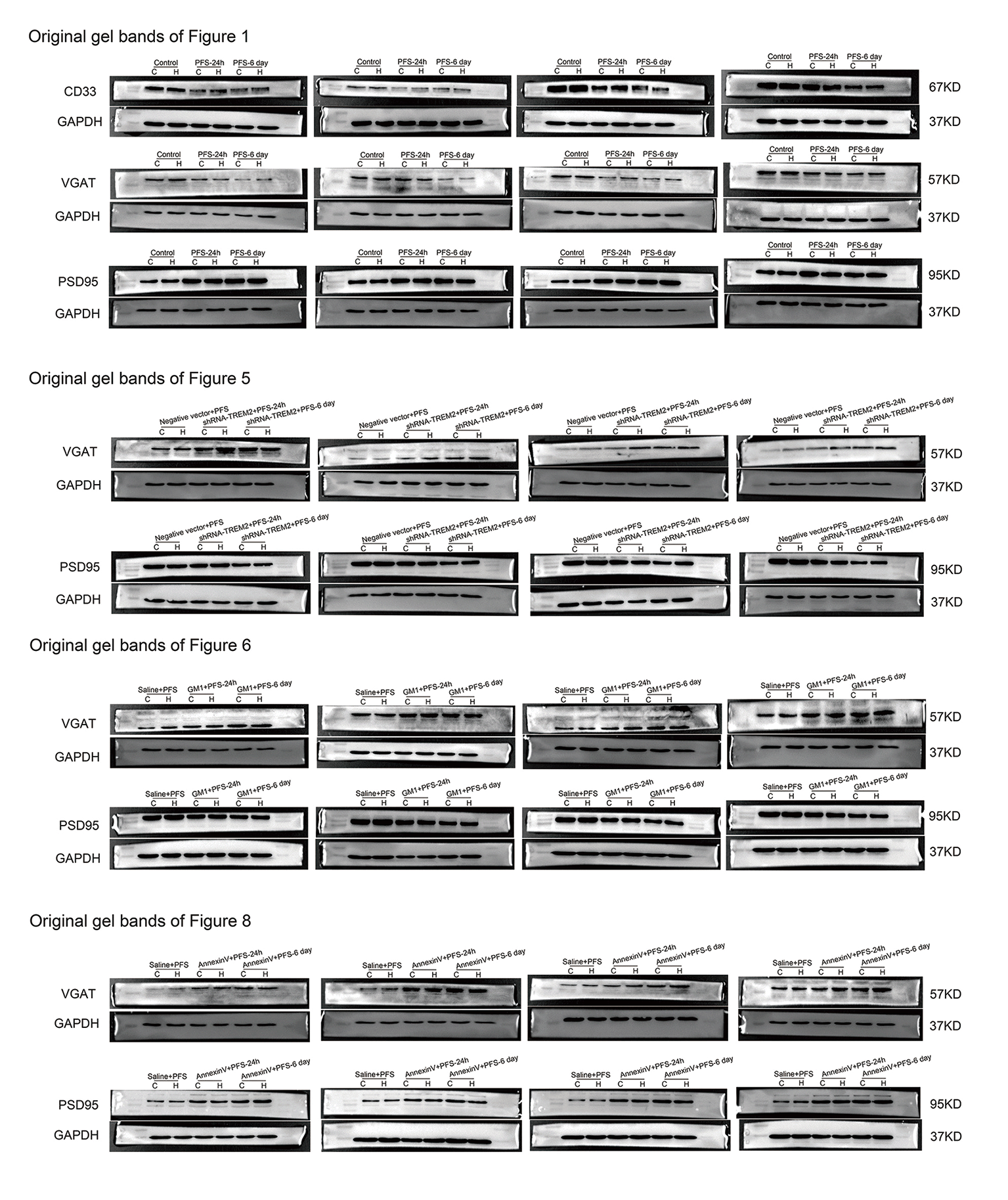

Supplement: Supplementary file 2 — Original gel bands of Figures 1 [file 41420_2026_3118_MOESM2_ESM.png]

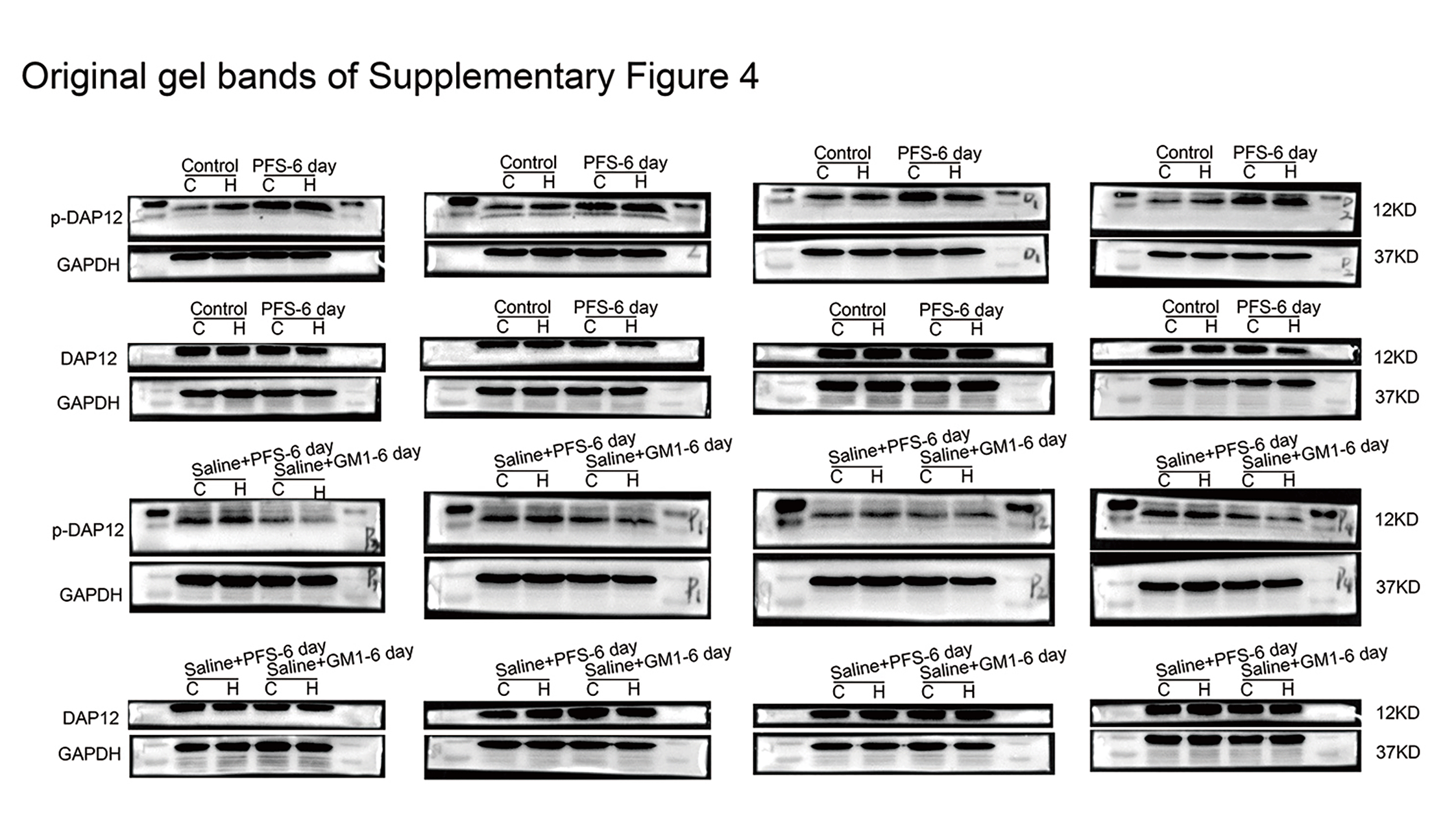

Supplement: Supplementary file 3 — Original gel bands of Figures 2 [file 41420_2026_3118_MOESM3_ESM.png]

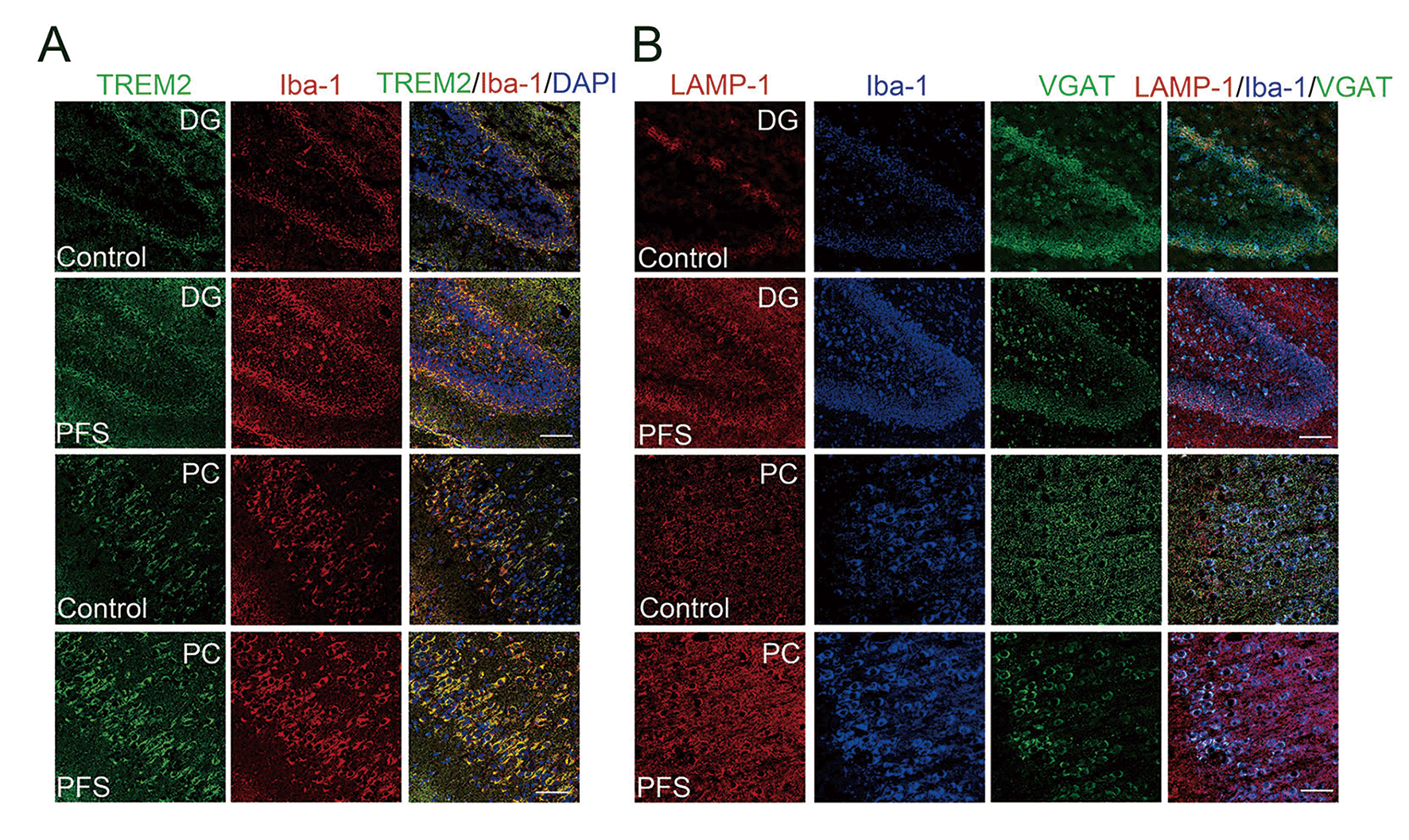

Supplement: Supplementary file 4 — Supplementary Figure 1 [file 41420_2026_3118_MOESM4_ESM.png]

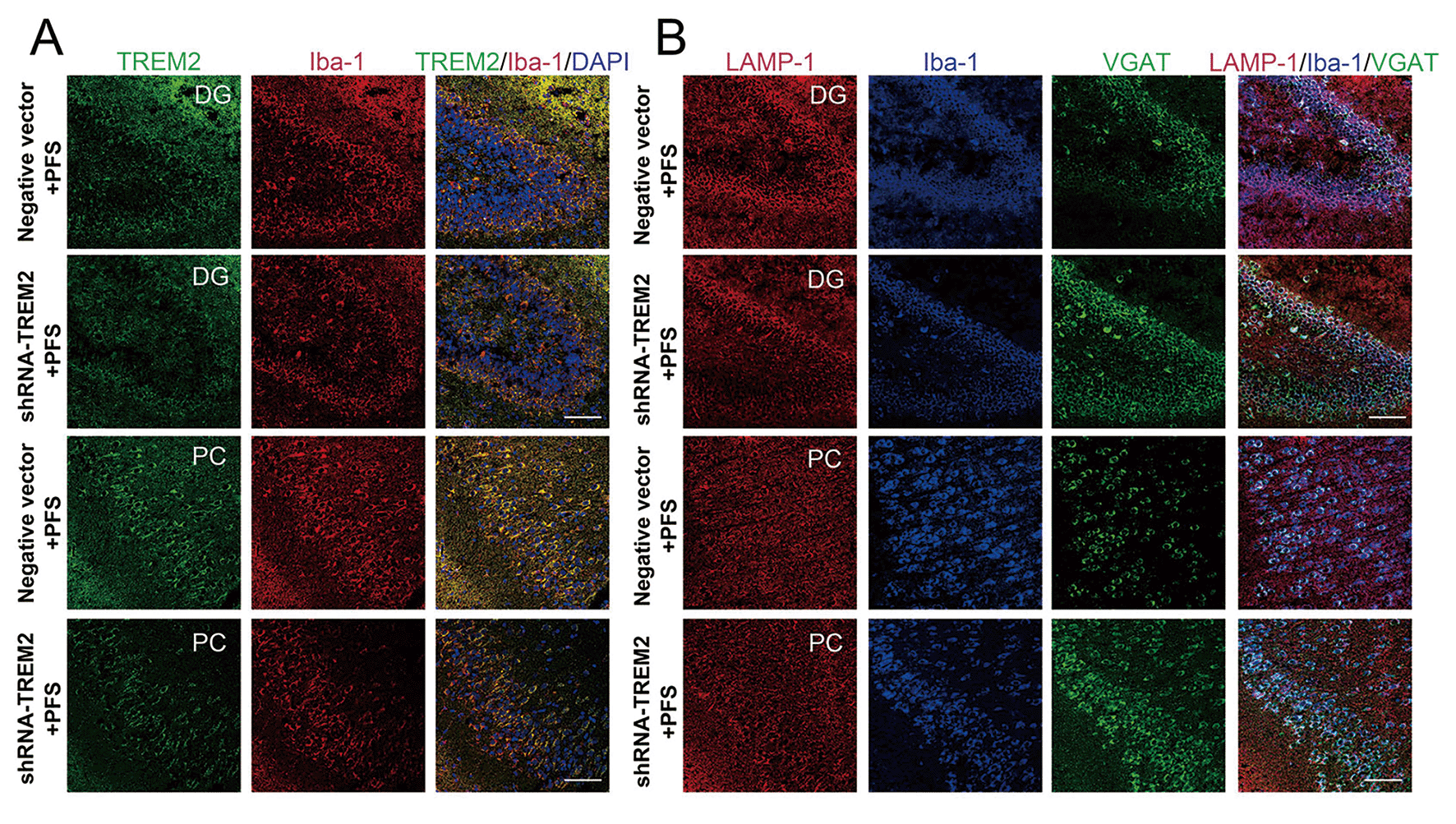

Supplement: Supplementary file 5 — Supplementary Figure 2 [file 41420_2026_3118_MOESM5_ESM.png]

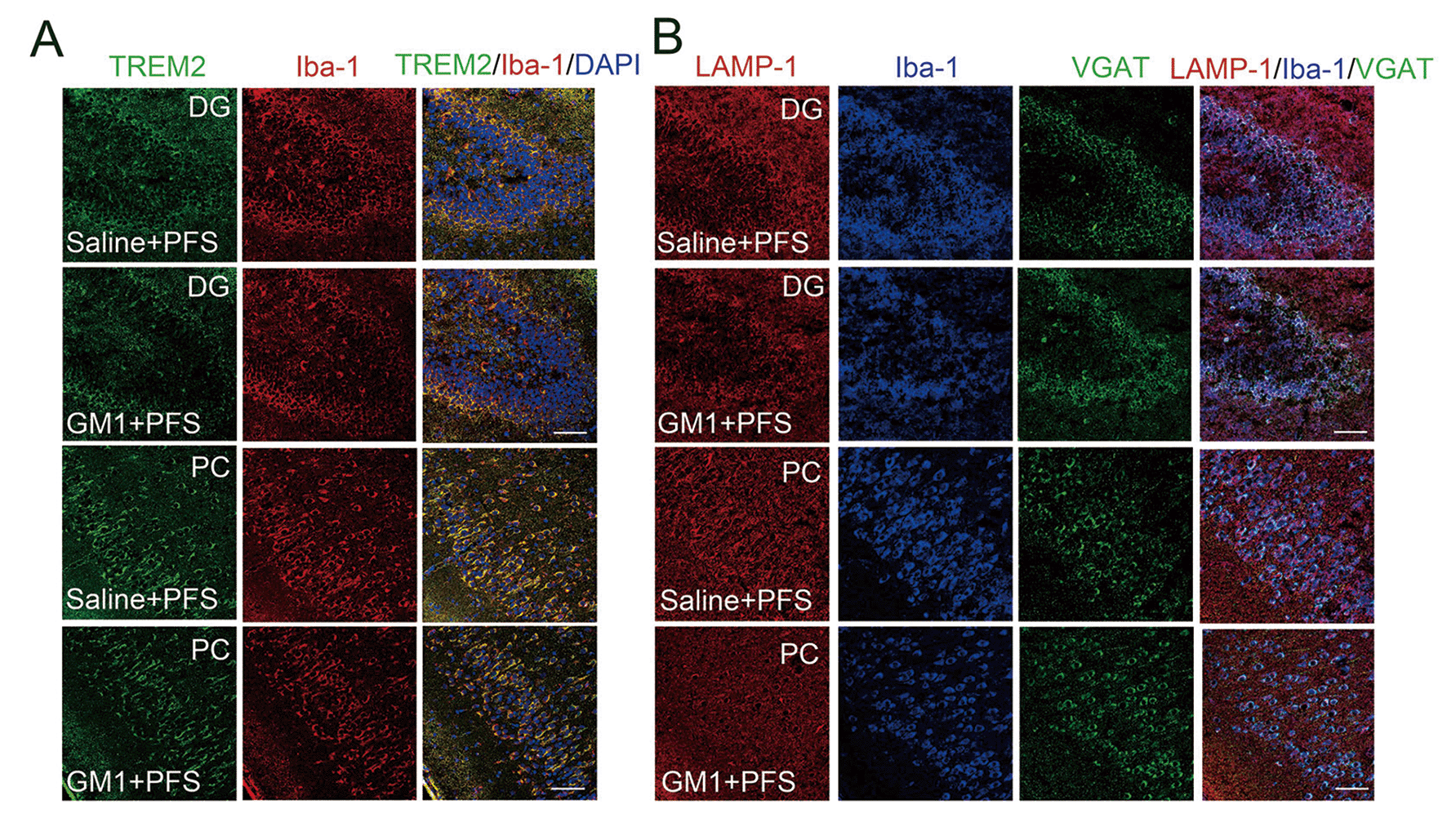

Supplement: Supplementary file 6 — Supplementary Figure 3 [file 41420_2026_3118_MOESM6_ESM.png]

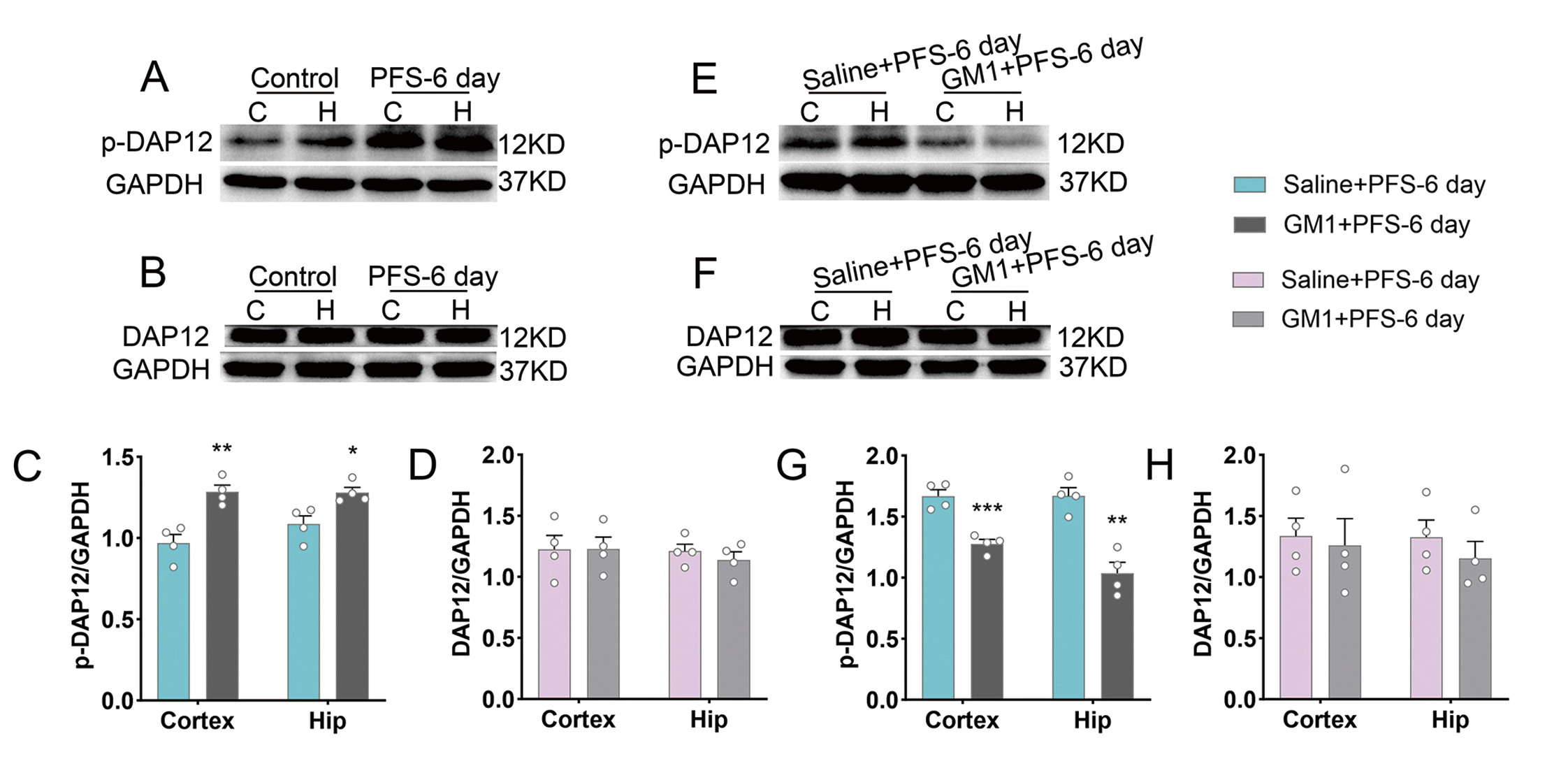

Supplement: Supplementary file 7 — Supplementary Figure 4 [file 41420_2026_3118_MOESM7_ESM.png]

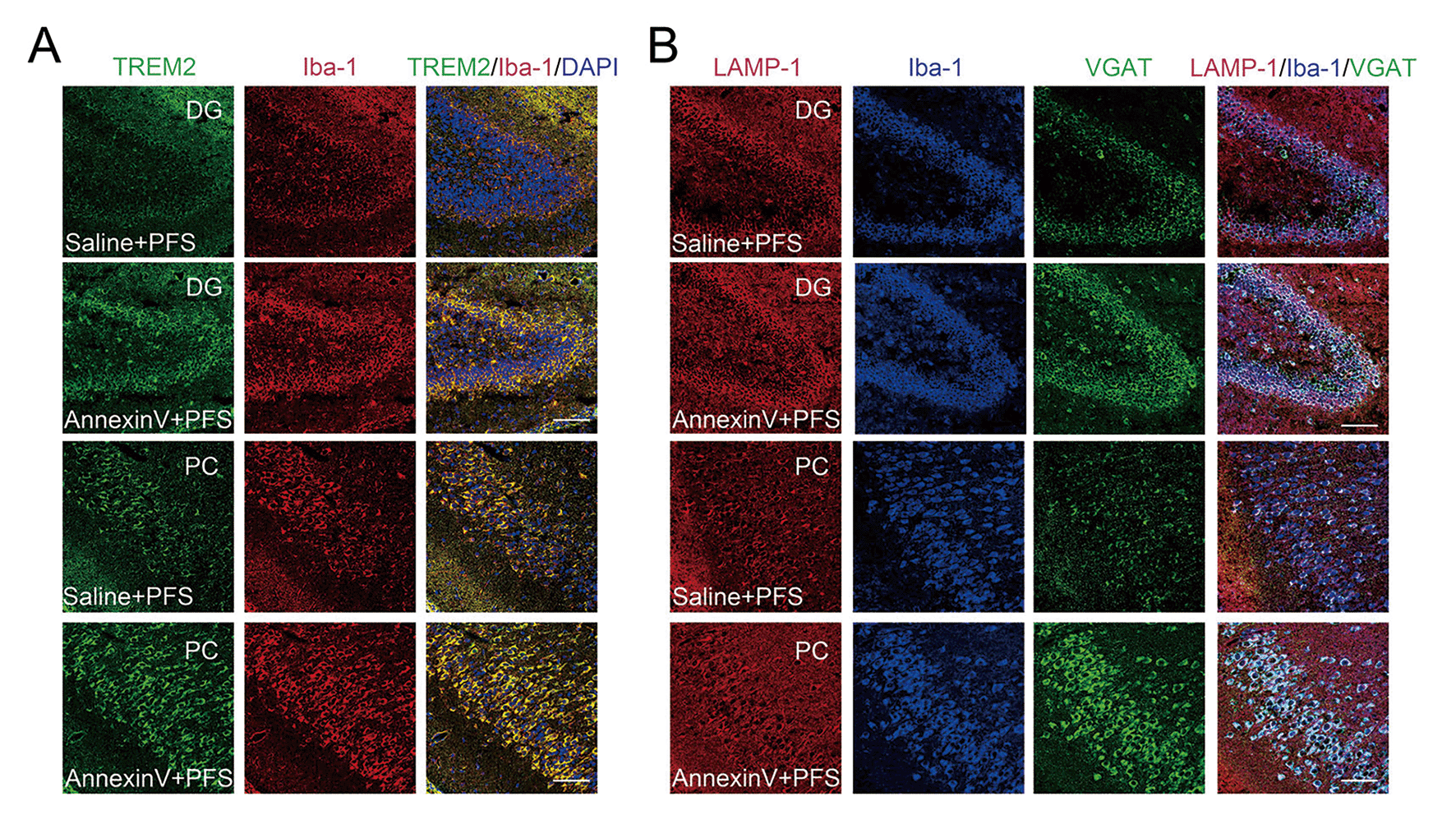

Supplement: Supplementary file 8 — Supplementary Figure 5 [file 41420_2026_3118_MOESM8_ESM.png]

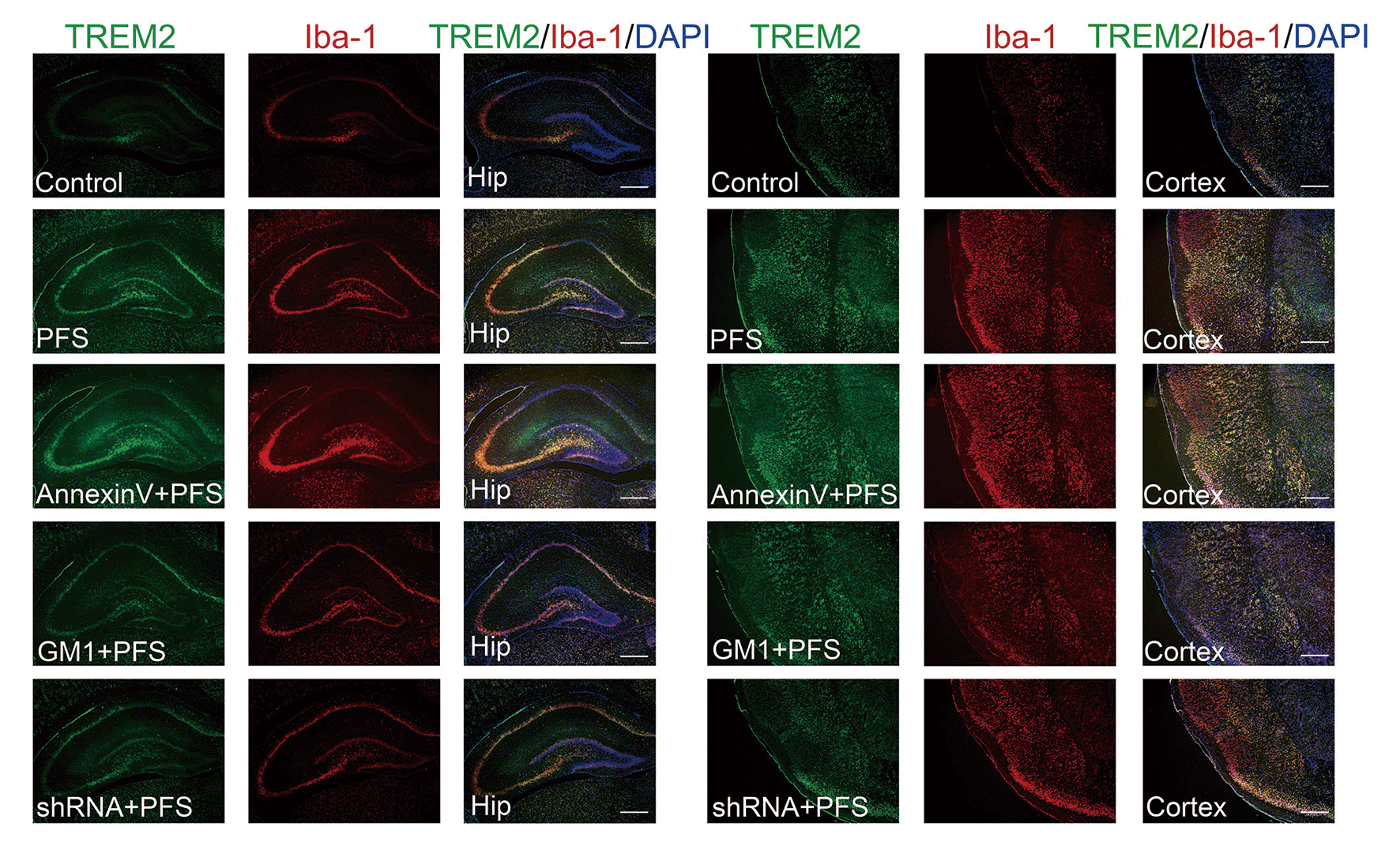

Supplement: Supplementary file 9 — Supplementary Figure 6 [file 41420_2026_3118_MOESM9_ESM.png]

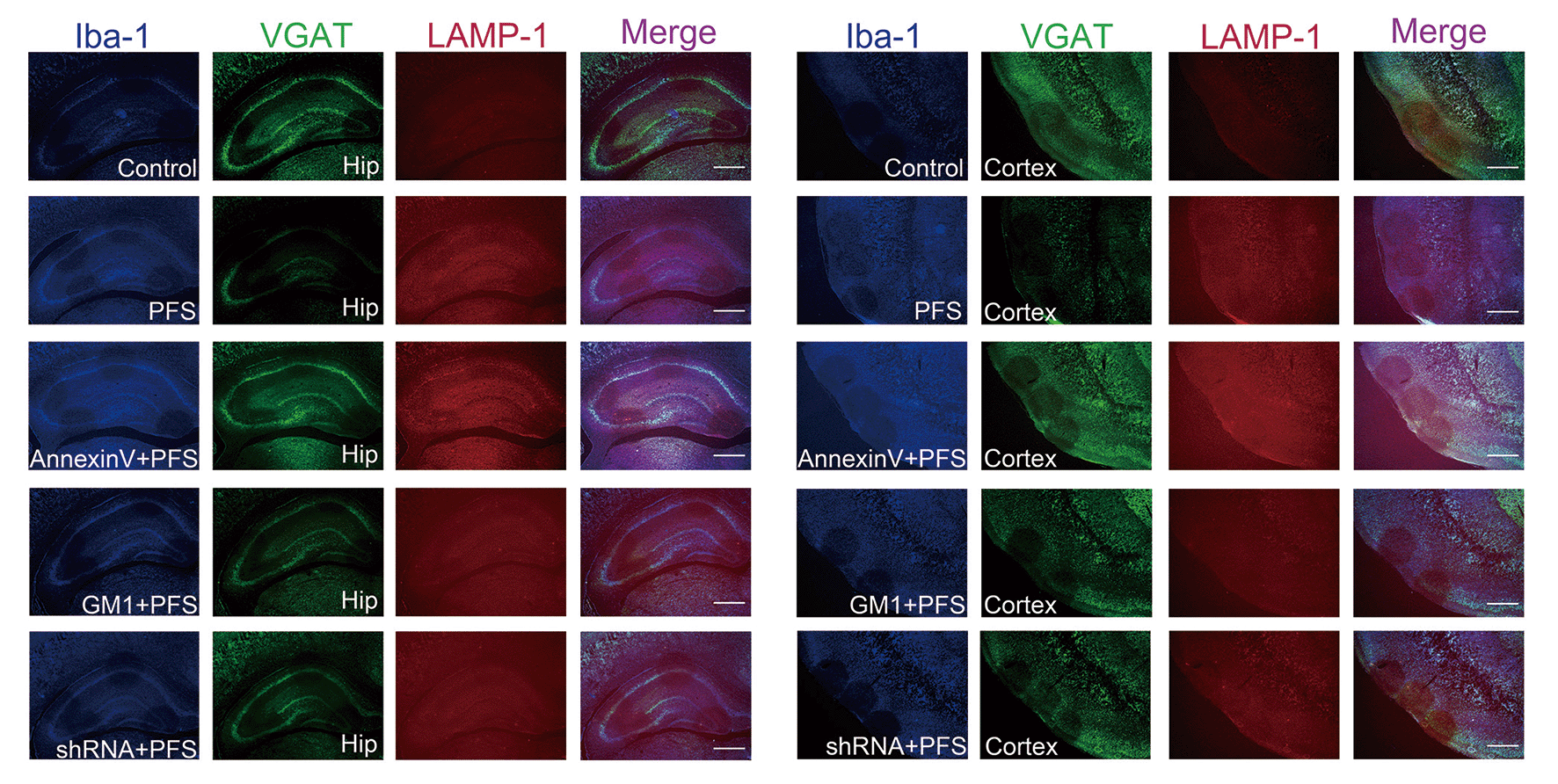

Supplement: Supplementary file 10 — Supplementary Figure 7 [file 41420_2026_3118_MOESM10_ESM.png]
